# Genome assembly of *Bougainvillia* cf. *muscus* (Cnidaria: Hydrozoa)

**DOI:** 10.1093/g3journal/jkaf110

**Published:** 2025-05-19

**Authors:** Aide Macias-Muñoz, Rebecca Varney, Eva Katcher, Maia Everhart, Todd H Oakley

**Affiliations:** Department of Ecology and Evolutionary Biology, University of California, Santa Cruz, Santa Cruz, CA 95064, USA; Department of Ecology, Evolution, and Marine Biology, University of California, Santa Barbara, Santa Barbara, CA 93106, USA; School of Biological Sciences, University of Nebraska–Lincoln, Lincoln, NE 68502, USA; Department of Ecology and Evolutionary Biology, University of California, Santa Cruz, Santa Cruz, CA 95064, USA; Department of Ecology and Evolutionary Biology, University of California, Santa Cruz, Santa Cruz, CA 95064, USA; Department of Ecology, Evolution, and Marine Biology, University of California, Santa Barbara, Santa Barbara, CA 93106, USA

**Keywords:** Cnidaria, synteny, repetitive elements, opsin, comparative genomics, phylogenetics

## Abstract

As one of just a handful of nonbilaterian animal phyla, Cnidaria are key to understanding genome evolution across Metazoa. Despite their importance and diversity, the genomes of most species in the phylum are unsequenced, due in large part to difficulties cultivating them in a laboratory. Here, we present a genome sequence of *Bougainvillia* cf. *muscus*, a hydrozoan with 4 marginal bulbs each containing 7 simple eyes (ocelli). This species appeared in our tanks from contamination. While we lacked sufficient samples for transcriptomic or functional studies, we were able to expand our knowledge of how the genome of this species compares to the few, better studied members of hydrozoans by investigating synteny to other cnidarians, repetitive element content, and phylogenetics and synteny of vision-related genes in this eyed species compared to eyeless relatives. The genome sequence consists of 350 contigs with an N50 of 10 Mb, a total genome length of 375.328 Mb, a BUSCO score of 90.1%, and predicted protein coding genes totaling 46,431. We found a high degree of macrosynteny conservation with *Hydra vulgaris*, *Hydractinia symbiolongicarpus*, and *Turritopsis rubra*. Repetitive elements make up 62% of this *Bougainvillia* genome. For vision-related genes, we identified 20 cnidarian opsins (cnidops) in *Bougainvillia* and found instances of gene duplication and loss in families associated with bilaterian eye development, phototransduction, and visual cycling. This high-quality, contiguous genome in an eyed hydrozoan will be a valuable resource for additional comparative genomic studies.

## Introduction

Comparative genomic studies are essential for understanding biodiversity, phylogenetic relationships, genome evolution, and the origins of organismal traits. With a growing number of high-quality genome assemblies, researchers can now examine how genomic changes contribute to animal evolution. For example, synteny analyses of ctenophore and sponge genomes have clarified deep animal relationships (Schultz *et al*. 2023), and similar methods have linked genomic changes to key events in vertebrate and annelid evolution (Simakov *et al*. 2020; Lewin *et al*. 2024). These studies demonstrate the power of new genomes to inform questions about evolution.

An especially important group for genomic comparisons is the phylum Cnidaria—which includes jellyfish, corals, and sea anemones. With ∼13,300 described species and as a sister group to Bilateria, cnidarians are central to understanding early animal evolution. Although recent efforts have expanded available cnidarian genomes (Santander *et al*. 2022), most genomic resources are from model organisms such as *Nematostella*, *Hydra*, and *Hydractinia* (Chapman *et al*. 2010; Kon-Nanjo *et al*. 2023; Zimmermann *et al*. 2023). As a result, much of the group's morphological and ecological diversity remains poorly sampled at the genomic level. Increasing the taxonomic breadth of cnidarian genomics will help reveal gene-level differences underlying their diverse forms and functions (Travert *et al*. 2023).

Cnidarians are also particularly valuable for studying the evolution of visual systems. Eyes evolved convergently at least 9 times within the phylum (Picciani *et al*. 2018; Miranda and Collins 2019). Some cnidarian eyes share developmental gene families with those of bilaterians, although often using different orthologs (Plachetzki *et al.* 2007; Koyanagi *et al*. 2008; Suga *et al.* 2008; Vöcking *et al*. 2022). One species, the box jellyfish *Tripedalia cystophora*, is the subject of diverse visual system research with published studies on morphology, gene expression, and visually guided behaviors (Nilsson *et al*. 2005; Garm *et al*. 2007; Garm, Oskarsson, and Nilsson 2011; Bielecki *et al*. 2014).

We intended to sequence the genome of *T. cystophora* when our culture unexpectedly produced small medusae that we sent for sequencing before discovering they were a contaminant of the hydrozoan *Bougainvillia* cf. *muscus* (herein referred to as *Bougainvillia*). Although we were unable to obtain polyps or additional medusae of the contaminant, thus preventing further analyses, we successfully generated a high-quality genome sequence. *Bougainvillia* belongs to Filifera, a hydrozoan suborder with few genomic resources (Kayal *et al*. 2015). Within Filifera, eyes likely evolved multiple times convergently, with *B. muscus* representing a distinct eye origin (named eye origin 4a) compared to other *Bougainvillia* (4b), *Turritopsis* (4d), and other Filifera (origin 5) (Picciani *et al*. 2018). To date, research on *Bougainvillia* has focused on morphology and ecology, with no high-quality genomic resource available (but see Edsinger *et al*. 2024).

**Fig. 1.**
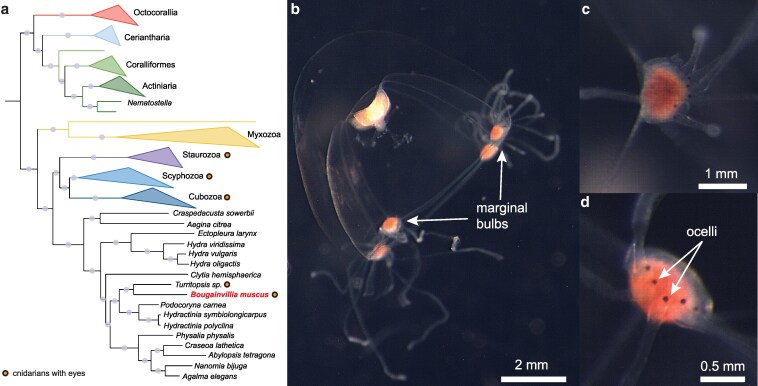
*B.* cf. *muscus* falls within Hydrozoa and has ocelli. a) Cnidarian phylogeny using 748 orthologs from DeBiasse *et al*. (2024) with *B.* cf. *muscus* added. Branches that have over 80% bootstrap support are labeled with light gray circles. Circles adjacent to species or class names represent cnidarians that have eyes. b) Photograph of a *Bougainvillia* medusa with marginal bulbs labeled. c, d) Zoomed in images of the marginal bulbs showing the ocelli, which are rounded, black, and located at the base of each tentacle. *Bougainvillia* have 7 ocelli in each of their 4 marginal bulbs.

Here, we present the genome of an eyed *Bougainvillia* species (Fig. 1). This assembly enables comparisons of synteny among hydrozoans, phylogenetic placement within Cnidaria, and investigation of genes involved in eye development and function. This assembly represents the best genomic resource to date for *Bougainvillia* and adds critical data for understanding cnidarian visual system evolution and genome diversity.

## Materials and methods

### DNA extraction

We obtained *Bougainvillia* from a tank of seawater at 25°C maintained at the University of California, Santa Barbara that also contained *T. cystophora* polyps. Water came from University of California, Santa Barbara's seawater system and the *T. cystophora* polyps came from a culture from Denmark, with *T. cystophora* originating from Puerto Rico or Florida. In September 2021, we began noticing many small medusae. We collected 15 free swimming medusae using plastic pipettes and washed them thoroughly with filtered seawater. We extracted DNA from fresh tissue using Circulomics Nanobind Tissue Big DNA Kit (NB-900-701-01; Circulomics via Pacific Biosciences, Menlo Park, CA, USA) skipping the tissueRuptor step.

### Data processing

The University of California, Irvine Genomics High Throughput Facility made PacBio HiFi low input libraries from our extracted DNA and sequenced them on 2 SMRT cells using PacBio Sequel II. We processed HiFi reads from BAM files using ccs v 6.4.0. In cell 1, 2,085,669 (46.63%) zero-mode waveguides passed filter and 1,982,604 (48.63%) in cell 2. We used Bam2fastq v. 1.3.0 to combine the sequencing data from the 2 cells and to extract it as fastq files. We generated a genome assembly using hifiasm (Cheng *et al*. 2021) and then determined completeness and contiguity using BUSCO v. 5.3.2 -l metazoa and BBmap v. 38.96 (Bushnell *et al*. 2014; Simão *et al*. 2015). To check for potential contaminants, we used BlobToolKit v. 4.2.1 and removed outlier contigs with no overlap to Cnidaria and with GC content < 0.3 and > 0.5 (Challis *et al*. 2020). We used BRAKER3 (Gabriel *et al*. 2024) to annotate the final assembly using protein models from *Hydra vulgaris* (Chapman *et al*. 2010), *Clytia hemisphaerica* (Leclère *et al*. 2019), *Hydractinia echinata*, and *Hydractinia symbiolongicarpus* (Schnitzler *et al*. 2024) as input.

### Species identification

We initially identified the medusae as *B. muscus* from morphological characters listed in the World Register of Marine Species (WoRMS Editorial Board 2024). After assembling the genome, we extracted sequences for Cytochrome c oxidase I (COI), 18S, 16S, and 28S ribosomal RNA genes and compared them to available sequences in NCBI via BLAST (Supplementary Table 1). The top hit was *B. muscus*. We then downloaded sequences of *B. muscus* COI, 18S, 16S, and 28S from NCBI and did reciprocal BLAST to the assembled genome. The sequences had an *e*-value of 0 suggesting a very close, if not exact match. For further validation, we downloaded COI, 16S, 18S, and 28S sequences for available *Bougainvillia* species from NCBI and generated phylogenetic trees using MAFFT to align sequences and iqtree2 with settings -m MFP -B 1000 -alrt 1000 -T 8 (Supplementary Fig. 1).

### Cnidarian species phylogeny

We obtained sequences for 748 genes previously aligned across cnidarians from DeBiasse *et al*. (2024). We used hmm2aln.pl (https://github.com/josephryan/hmm2aln.pl) to identify sequences that matched each of these alignments in *Bougainvillia*. We then filtered for the best match (longest sequence with least gaps), ran MAFFT v. 7.520 (default parameters) and Gblocks v0.91b (-b2 = 10 -b3 = 10 -b4 = 5 -b5 = a), and concatenated the matrix (see ‘*Data availability*’). We generated a phylogenetic tree from the concatenated matrix using iqtree2 v. 2.2.2.6 -nt AUTO -bb 1000 -m TEST and then used iTOL v6 to visualize, root (outgroups Bilateria, Ctenophora, and Porifera), and annotate the tree. We also generated a tree from concatenated alignments prior to Gblocks using the same model.

### Macrosynteny and repetitive sequences

To characterize synteny conservation across hydrozoans, we investigated macrosynteny between our final *Bougainvillia* genome assembly and the *Turritopsis*, *Hydra*, and *Hydractinia* chromosome-level genome assemblies (Simakov *et al*. 2020; Kon-Nanjo *et al*. 2023; Dong *et al*. 2024). We downloaded the *Turritopsis rubra* (GCA_039566895.2) and *H. vulgaris* v3 (GCF_022113875.1) genomes from NCBI and the *H. symbiolongicarpus* v2.0 (https://doi.org/10.6084/m9.figshare.22126232.v1) genome from Figshare (University of Vienna 2022; Kon-Nanjo *et al*. 2023; Yantai Institute of Coastal Zone Research 2024). We used odp with a minimum scaffold size of 1 Mb for the comparisons (Schultz *et al*. 2023). To estimate the degree of macrosynteny conservation, we calculated the number of one-to-one orthologs in homologous locations and divided by the total number of orthologs in the odp map (Wang *et al*. 2017; Li *et al*. 2022).

To identify repetitive sequences, we first generated a custom library using default parameters in RepeatModeler v. 2.0.5 (Flynn *et al*. 2020) and then used it to identify repeats in the genome assembly with RepeatMasker v. 4.1.5 (http://www.repeatmasker.org) (Smith *et al*. 2013-2015).

### Vision-related phylogenetic trees

To investigate the phylogenetic relationships of vision-related genes across cnidarians, we downloaded publicly available transcriptomes from NCBI using NCBI's datasets command-line tools (Supplementary Table 2) (Sanders and Cartwright 2015; Chen *et al*. 2016; Hasegawa *et al*. 2016; Yunfeng *et al*. 2017; Lewis Ames *et al*. 2016; Kayal *et al*. 2018; Khalturin *et al*. 2019; Ying *et al*. 2019; Kupaeva *et al*. 2020; Xia *et al*. 2020; Ortiz González *et al*. 2021). We used TransDecoder v. 5.7.1 to extract coding and peptide sequences and cd-hit v. 4.8.1 with parameters -c 0.9 -n 5 to remove redundant transcripts (Li and Godzik 2006; Fu *et al*. 2012). We used OrthoFinder v. 2.5.5 to identify orthogroups among all datasets (Emms and Kelly 2019). We aligned sequences for candidate genes using MAFFT v. 7.520 and generated trees using iqtree2 v. 2.2.2.6 -m MFP -B 1000 -alrt 1000 -T 8 (Katoh and Standley 2013; Minh *et al*. 2020 ). We used iTOL v6 to annotate our final tree files, rooted at midpoint (Letunic and Bork 2024). For the opsin tree, we downloaded opsin sequences from (McCulloch *et al*. 2023) and searched for similar sequences in the *Bougainvillia* genome assembly using command-line BLAST+. We extracted sequences for the top hits and searched against the NCBI database to confirm opsin annotation. We added all *Bougainvillia* sequences that matched an opsin annotation to the opsin fasta file from (McCulloch *et al*. 2023) and used them as input for MAFFT and iqtree2. We rooted the tree using *Trichoplax* placopsins (Feuda *et al*. 2012; Fleming *et al*. 2020).

## Results and discussion

### *B.* cf. *muscus* morphology and phylogeny

*Bougainvillia* medusae were ∼6 mm in height and width and had 4 red structures at the corners of the bell. Higher magnifications revealed that these structures were tentacle bulbs, each with 7 simple eyes (ocelli) (Fig. 1). Medusae had a branching manubrium common to the genus (Fig. 1). Morphological identification coupled with 18S/COI/16S alignments and phylogenetic analyses all supported the species identification as *B. muscus* (Supplementary Fig. 1). A more data rich phylogenetic placement based on genes from across the genome was consistent with this identification, with *Bougainvillia* falling as sister to *Turritopsis* to form the Pseudothecata (Fig. 1; Supplementary Fig. 2) (Mendoza-Becerril *et al*. 2018).

### Genome assembly

The final *Bougainvillia* genome assembly consisted of 350 contigs with an N50 of 10 Mb, max length of 26.349 Mb, and a total genome length of 375,328,287 bases (Table 1). Diverse cnidarian species including *Hydra*, *Nematostella*, *Hydractinia*, and *Clytia* have 15 chromosomes (Leclère *et al*. 2019; Simakov *et al*. 2022; Kon-Nanjo *et al*. 2023; Zimmermann *et al*. 2023), so we might expect a similar number in *Bougainvillia*. While we did not recover 15 chromosomes, the number of contigs is comparable to those of the *Hydractinia* genome before scaffolding with Hi-C (Kon-Nanjo *et al*. 2023). The *Bougainvillia* genome was smaller than those of other hydrozoans. The *Hydractinia* genome is ∼483 Mb, the *Clytia* genome is ∼445 Mb, the *H. vulgaris* 105 strain is ∼819 Mb, *Hydra* AEP is ∼901 Mb, and *Hydra oligactis* is ∼1,274 Mb (Leclère *et al*. 2019; Simakov *et al*. 2022; Cazet *et al*. 2023; Kon-Nanjo *et al*. 2023; Zimmermann *et al*. 2023). The GC content of the *Bougainvillia* genome assembly was 36.21%, indicating it is AT-rich like other hydrozoans (Chapman *et al*. 2010; Schnitzler *et al*. 2024).

**Table 1.** Genome assembly statistics.

Genome feature	Value
Cell 1 bases read	474.02 Gb
Cell 1 polymerase reads	4,472,747
Cell 2 bases read	438.52 Gb
Cell 2 polymerase reads	4,076,913
Genome length	375.33 Mb
Contig number	350
Contig N50	10 Mb
Contig L50	16.62 Mb
Contig N90	22 Mb
Contig L90	6.465 Mb
Max contig length	26.35 Mb
GC	36.21%
BUSCO	90.10%
Genes	46,431

The final *Bougainvillia* assembly had a complete BUSCO score of 90.1%. Using BRAKER3, we identified 46,431 predicted protein-coding genes (Table 1). This number is larger than those of the other cnidarian genomes, which have ∼20–30 thousand genes (Gold *et al*. 2019; Leclère *et al*. 2019; Cazet *et al*. 2023; Zimmermann *et al*. 2023). The number of predicted genes in our assembly is likely due to splice variants included as multiple separate gene models. To collapse similar sequences into single gene models, we clustered sequences using cd-hit at cutoff 0.9, yielding 33,515 predicted genes (BUSCO 90.8%). To our knowledge, this is the most contiguous assembly for a hydrozoan outside of *Hydra*, *Hydractinia*, and *Turritopsis*.

### Synteny

**Fig. 2.**
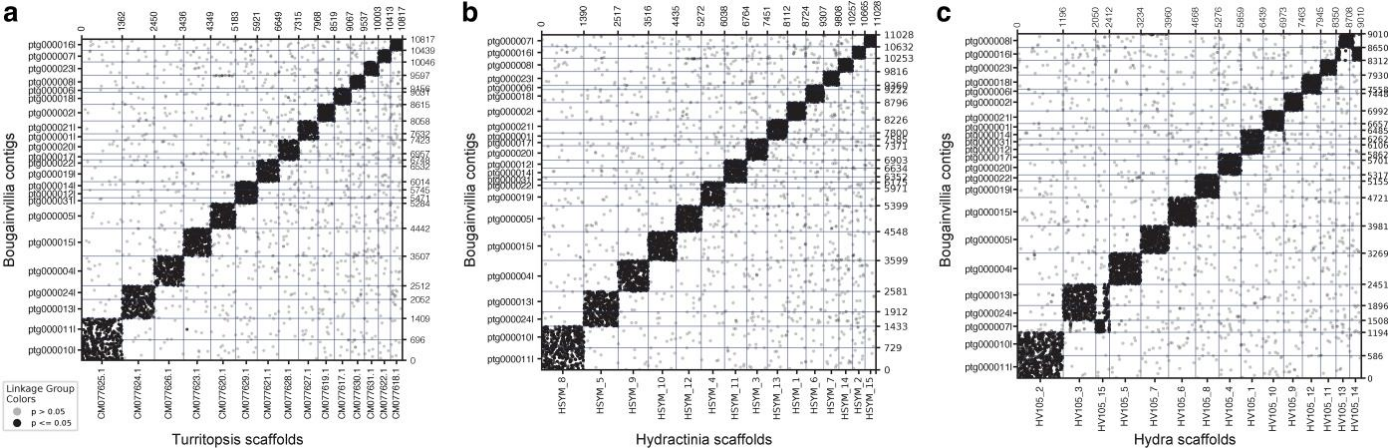
*Bougainvillia* is syntenic with other hydrozoans. a) Oxford dot plots showing the location of 10,897 orthologous genes in 23 *Bougainvillia* contigs and 15 *T. rubra* scaffolds. b) Oxford dot plots showing the location of 11,029 orthologous genes in 23 *Bougainvillia* contigs and 15 *H. symbiolongicarpus* scaffolds. c) Oxford dot plots showing the location of 9,011 orthologous genes in 23 *Bougainvillia* contigs and 15 *H. vulgaris* scaffolds.

Due to the limited number of high-quality genomes within Cnidaria, the degree of macrosynteny across the phylum remains understudied. However, previous studies in cnidaria suggest some phylogenetic signal in macrosynteny (Kon-Nanjo *et al*. 2023; Zimmermann *et al*. 2023). For example, *Nematostella* has a high degree of macrosynteny conservation with a closely related sea anemone (*Scolanthus*) but less conservation with *Hydra* and *Xenia* (Zimmermann *et al*. 2023). To determine the level of macrosynteny conservation between *Bougainvillia* and other hydrozoans, we compared our assembly to the chromosome-level assemblies of *T. rubra*, *H. vulgaris* 105 strain, and *H. symbiolongicarpus*. *Hydra* and *Hydractinia* have high macrosynteny conservation, with 2 potential translocations (Kon-Nanjo *et al*. 2023). Here, we found a high degree of macrosynteny conservation in *Bougainvillia* compared to *Turritopsis* and *Hydractinia* (Fig. 2). We did not detect any obvious genome rearrangements and calculated the conservation index to be 0.866 and 0.863, respectively.

Between *Bougainvillia* and *Hydra*, we found macrosynteny to be mostly conserved, with some potential chromosome rearrangements (Fig. 2b). Firstly, there was a potential translocation between *Hydra* chromosomes 3 and 15 and *Bougainvillia* contigs ptg000013l, ptg000024l, and ptg000007l (Fig. 2). *Bougainvillia* contigs ptg000013l and ptg000024l were syntenic to most of *Hydra* chromosome 3 and part of chromosome 15. Meanwhile, ptg000007l was syntenic to a small part of *Hydra* chromosome 3 and syntenic to 2 pieces of chromosome 15. The other potential genomic rearrangement was ptg000016l, which was syntenic to parts of *Hydra* chromosomes 13 and 14. These translocations are consistent with the macrosynteny analysis between *Hydra* and *Hydractinia* supporting that the translocation occurred after the divergence of *Hydra* and the *Hydractinia* and *Bougainvillia* clade. The conservation index between *Bougainvillia* and *Hydra* was 0.838. Our results suggest that *Bougainvillia* is more syntenic with *Turritopsis* and *Hydractinia* than with *Hydra*, reflecting their phylogenetic relationships.

### Repetitive elements

**Fig. 3.**
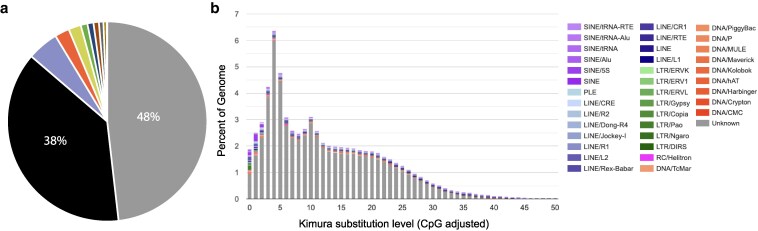
The *Bougainvillia* genome is highly repetitive. a) Pie chart showing the percent of the genome made up of unclassified repetitive elements (gray), LINE (blue), DNA transposons (orange), small RNA (yellow), SINE (green), LTR (dark blue), Penelope (magenta), rolling circles (dark gray), simple repeats (brown), and unmasked (black). b) Repeat landscape of the *Bougainvillia* genome assembly.

Repetitive elements make up ∼61% of the *H. symbiolongicarpus* genome and ∼70% of the *H. vulgaris* genome (Cazet *et al*. 2023; Kon-Nanjo *et al*. 2023). A recent study identified active TEs (Transposable elements) driving variation in genome size between 2 *H. vulgaris* strains (Kon-Nanjo *et al*. 2024). In addition, an in-depth characterization of TEs in *Hydractinia* found the subfamily Helitron is responsible for a recent expansion of repetitive elements (Kon *et al*. 2025). To compare repetitive content in *Bougainvillia* to other hydrozoans, we classified the number of repetitive elements in our genome assembly. Similar to *Hydractinia*, repetitive elements made up 61.78% of the *Bougainvillia* genome, with ∼48% being unclassified or unknown (Fig. 3a; Table 2). Following unclassified elements, retroelements in the LINE group made up the next largest percent of repetitive elements, 5% of the genome. Unlike in *Hydractinia*, whose sequence divergence analysis suggested 2 conspicuous episodes of repetitive element expansions, in *Bougainvillia* expansion of repetitive elements appears to be more continuous but also shows 2 peaks of repeat expansions (Fig. 3b).

**Table 2.** RepeatMasker output for the *Bougainvillia* genome.

	Number of elements	Length occupied (bp)	Percentage of sequence
Retroelements	87,012	26,807,637	7.14
SINEs	12,334	4,105,380	1.09
Penelope	10,802	3,305,874	0.88
LINEs	65,407	19,011,721	5.07
CRE/SLACS	748	360,091	0.1
L2/CR1/Rex	18,383	4,904,952	1.31
R1/LOA/Jockey	347	47,488	0.01
R2/R4/NeSL	997	714,097	0.19
RTE/Bov-B	14,312	4,773,644	1.27
L1/CIN4	1,110	216,104	0.06
LTR elements	9,271	3,690,536	0.98
BEL/Pao	1,448	1,148,511	0.31
Ty1/Copia	160	124,659	0.03
Gypsy/DIRS1	3,420	1,781,405	0.47
Retroviral	3,792	352,487	0.09
DNA transposons	102,744	8,895,639	2.37
hobo-Activator	2,781	353,672	0.09
Tc1-IS630-Pogo	3,510	437,421	0.12
En-Spm	—	—	0
MULE-MuDR	211	32,456	0.01
PiggyBac	51	28,969	0.01
Tourist/harbinger	1,042	264,753	0.07
Other	12,370	870,658	0.23
Rolling circles	17,411	2,737,491	0.73
Unclassified	1,322,062	181,344,581	48.32
Total interspersed repeats		220,353,731	58.71
Small RNA	7,753	7,589,974	2.02
Satellites	—	—	0
Simple repeats	41,673	2,003,055	0.53
Low complexity	6,601	306,921	0.08
Total bases masked	—	231,870,804	61.78

### Vision-related genes

#### Opsins

**Fig. 4.**
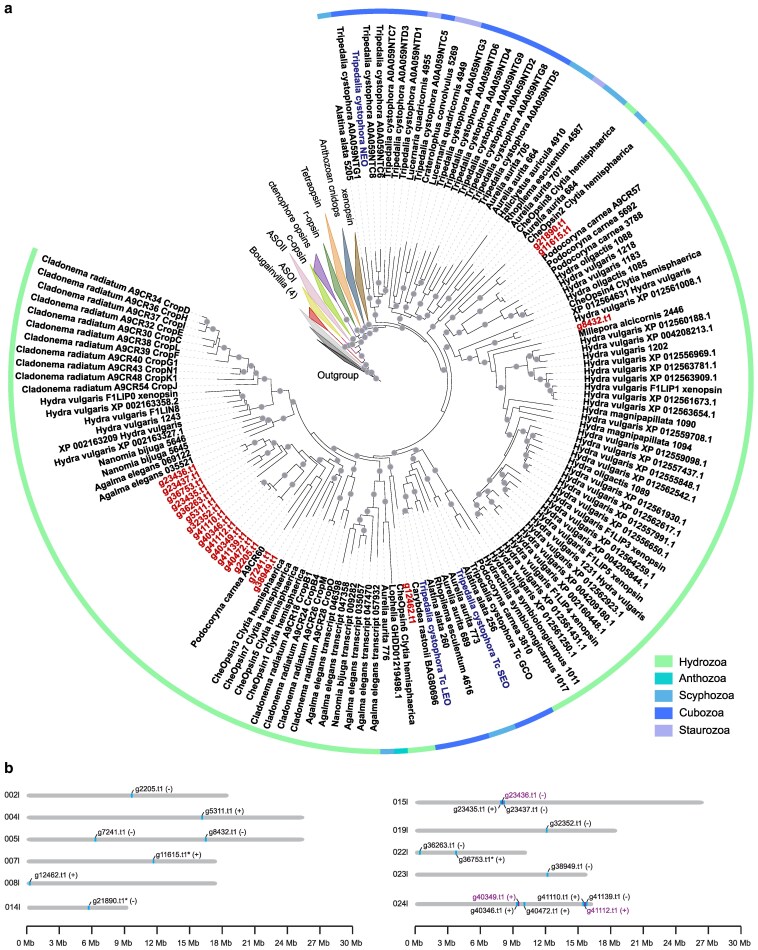
*Bougainvillia* opsin phylogeny and genome location. a) Cnidarian opsin (cnidops) phylogeny. *Bougainvillia* opsins are labeled in red; they are the shortes genes that begin with the letter g and end with .t1. *Tripedalia* opsins known to be expressed in eyes are shown in blue. Branches that have over 80% bootstrap support are labeled with circles. b) Schematic of cnidops locations on the genome. Contigs are shown as gray bars, and cnidops locations are colored and labeled (typically blue). Genes that are located close to each other are labeled in purple. Orientation is shown after the gene name in parentheses. Genes with asterisks after their name are those that have introns.

Opsin genes encode proteins that are typically used to detect light. Since *Bougainvillia* has ocelli, we examined opsin gene content. We identified a total of 24 predicted opsin-like genes in *Bougainvillia* (Fig. 4; Supplementary Table 3). On the opsin phylogenetic tree, we found 4 of the genes grouped outside of the cnidops (cnidarian opsin) group and were not opsins based on additional BLAST analyses, but rather other G protein-coupled receptors (Fig. 4). The remaining 20 genes were cnidops. This is comparable to the 17 opsins identified in *Tripedalia* (Liegertová *et al*. 2015). This number is expected for Medusozoa, which have a smaller diversity of opsins compared to Anthozoa (McCulloch *et al*. 2023).

Of the 20 cnidops genes, 2 group together on the phylogenetic tree (*g21890.t1* and *g11615.t1*) and are closely related to *Clytia* opsin *CheOpsin2*. Another gene, *g8432.t1*, groups with a *Hydra* cnidops group. One *Bougainvillia* cnidops *g12462.t1* groups together with *CheOpsin6*. The remaining cnidops form a clade together with a *Podocoryna* A9CR60 and *Clytia* CheOpsins 1, 5, 7, 3 (Fig. 4). In terms of phylogenetic position, none of the *Bougainvillia* opsin genes were orthologs of cnidops expressed in *Tripedalia* eyes (Bielecki *et al*. 2014; Garm *et al*. 2022) (Fig. 4). This suggests *Bougainvillia* may be using a nonorthologous opsin for visual function, indicating probable paralog switching. The 20 *Bougainvillia* cnidops genes are distributed across 11 chromosomes and all but 3 lack introns (Fig. 4; Supplementary Table 2). This is consistent with medusozoan opsins being intronless (Plachetzki *et al.* 2007; Liegertová *et al*. 2015). We also identified some cnidops that are located in tandem. This is the case for 3 *Bougainvillia* cnidops on contig ptg000015l and 2 pairs of genes on ptg000024l. Interestingly, the 6 cnidops genes on ptg000024l group together on the phylogenetic tree indicating similarity in gene sequence, including the 2 pairs of genes found in tandem (Fig. 4). The structure and location of the *Bougainvillia* opsins suggest molecular evolution by retrotransposition and tandem duplication similar to *Hydra* and *Nematostella* opsins (Macias-Munõz *et al.* 2019; McCulloch *et al*. 2023).

#### Other vision-related genes

To investigate the molecular evolution of other candidate vision-related genes in *Bougainvillia*, we identified genes similar to those involved in eye development, phototransduction, and visual cycling from model organisms. We generated phylogenetic trees and counted the number of genes present in each gene family within the *Bougainvillia* genome, *T. cystophora* (a box jellyfish, which all have eyes) transcriptome, and *Hydra* (an eyeless hydrozoan) gene models (Table 3; Supplementary Figs. 3–23). The gene families BMP/GDF, Hedgehog, and Wnt function in visual system specification and have undergone duplications and expansions in many animal groups, so we expected to find differences in copy number. We found several genes to be present in *Bougainvillia* and *Tripedalia* but absent in eyeless *Hydra*. These included *dpp-like*, *wnt11-like*, *wnt4-like*, and *SIX1/2-like*. Dpp—or decapentaplegic—is necessary for eye patterning in *Drosophila* and can induce ectopic expression of photoreceptor cell differentiation (Pignoni and Zipursky 1997). SIX-family genes also play a role in retinal specification in *Drosophila*. For phototransduction, G-alpha subunits, adenylate cyclase (AC), and cyclic nucleotide-gated (CNG) channels have been identified as probable components of cnidarian phototransduction cascades (Plachetzki *et al.* 2007; Koyanagi *et al*. 2008), but diversity across clades is possible (Vöcking *et al*. 2022). For phototransduction and visual cycling genes, we found some potential instances of gene duplication or loss across the 3 cnidarians. However, except for G-alpha-s (Koyanagi *et al*. 2008), the specific functions of these candidate genes in vision have never been demonstrated in any cnidarian. Future studies of eye development and phototransduction in cnidarians will reveal whether these genes play functional roles in eye patterning and light detection.

**Table 3.** Summary of vision-related gene copy numbers.

	*Hydra*	*Tripedalia*	*Bougainvillia*	
Visual system specification				
BMP/GDF	7	9	8	Supplementary Fig. 3
Hedgehog (Hh)	3	1	2	Supplementary Fig. 4
Wnt	10	11	12	Supplementary Fig. 5
Retinal determination				
Eyes absent	1	1	1	Supplementary Fig. 6
Pax	3	3	3	Supplementary Fig. 7
SIX	2	4	4	Supplementary Fig. 8
Phototransduction				
Galphai	1	1	1	Supplementary Fig. 9
Galphas	1	2	2	Supplementary Fig. 10
Galphaq	1	1	1	Supplementary Fig. 11
Gbeta	3	2	3	Supplementary Fig. 12
CNG	4	2	3	Supplementary Fig. 13
TRPC	1	1	1	Supplementary Fig. 14
PLC	6	2	1	Supplementary Fig. 15
AC13E	2	1	1	Supplementary Fig. 16
AC2/5-like	2	2	2	Supplementary Fig. 17
GC	3	7	4	Supplementary Fig. 18
Arrestin	1	1	1	Supplementary Fig. 19
Visual cycling				
GRK	1	1	1	Supplementary Fig. 20
Rhk	1	1	1	Supplementary Fig. 21
SEC14	2	3	5	Supplementary Fig. 22
Phosphodiesterase	5	3	3	Supplementary Fig. 23

## Conclusions

We generated a high-quality and contiguous genome for a hydrozoan with a visual system consisting of multiple ocelli. The *Bougainvillia* genome assembly consists of 350 contigs with an N50 of 10 Mb and a total genome length of 375 Mb. Although the *Bougainvillia* genome is smaller than other hydrozoans, macrosynteny is highly conserved with *H. symbiolongicarpus* and *H. vulgaris*, supporting synteny across hydrozoans. Similar to *Hydractinia*, ∼62% of the *Bougainvillia* genome was made up of repetitive elements. Exploration of vision-related genes identified candidate genes for future studies of eye development and light detection in cnidarians. This new hydrozoan genome is valuable for comparative studies in cnidarian and metazoan biology.

## Supplementary Material

jkaf110_Supplementary_Data

## Data Availability

Data associated with this genome were deposited to NCBI under BioProject PRJNA1215439. Raw sequencing read data are available in SRA under accessions SAMN46550779 and SAMN46550780, and circular consensus sequencing reads are available under accessions SRX27573578 and SRX27573579. The final genome assembly is available in GenBank under accession JBLHEM000000000 and figshare (https://doi.org/10.6084/m9.figshare.29064560). The final genome assembly and associated files are also available for download in Zenodo (10.5281/zenodo.14727809). All codes associated with this manuscript are available on GitHub (https://github.com/amm1623/Bougainvillia_genome) and Zenodo (10.5281/zenodo.14727809). For our synteny analyses, we obtained *Hydra vulgaris* v3 from NCBI (accession GCF_022113875.1), *Turritopsis rubra* from NCBI (GCA_039566895.2), and *Hydractinia symbiolongicarpus* v2.0 from figshare (https://doi.org/10.6084/m9.figshare.22126232.v2). Supplemental material available at G3 online.
