# Supplementary material for: Genome assembly of Bougainvillia cf. muscus (Cnidaria: Hydrozoa)
Source: G3 (Bethesda). 2025 May 19;15(7):jkaf110. doi: 10.1093/g3journal/jkaf110 (PMC12239608; doi:10.1093/g3journal/jkaf110)
Supplement: jkaf110_Supplementary_Data [file jkaf110_supplementary_data.zip › Supplemental_Figures_G3-2025-405923.pdf]

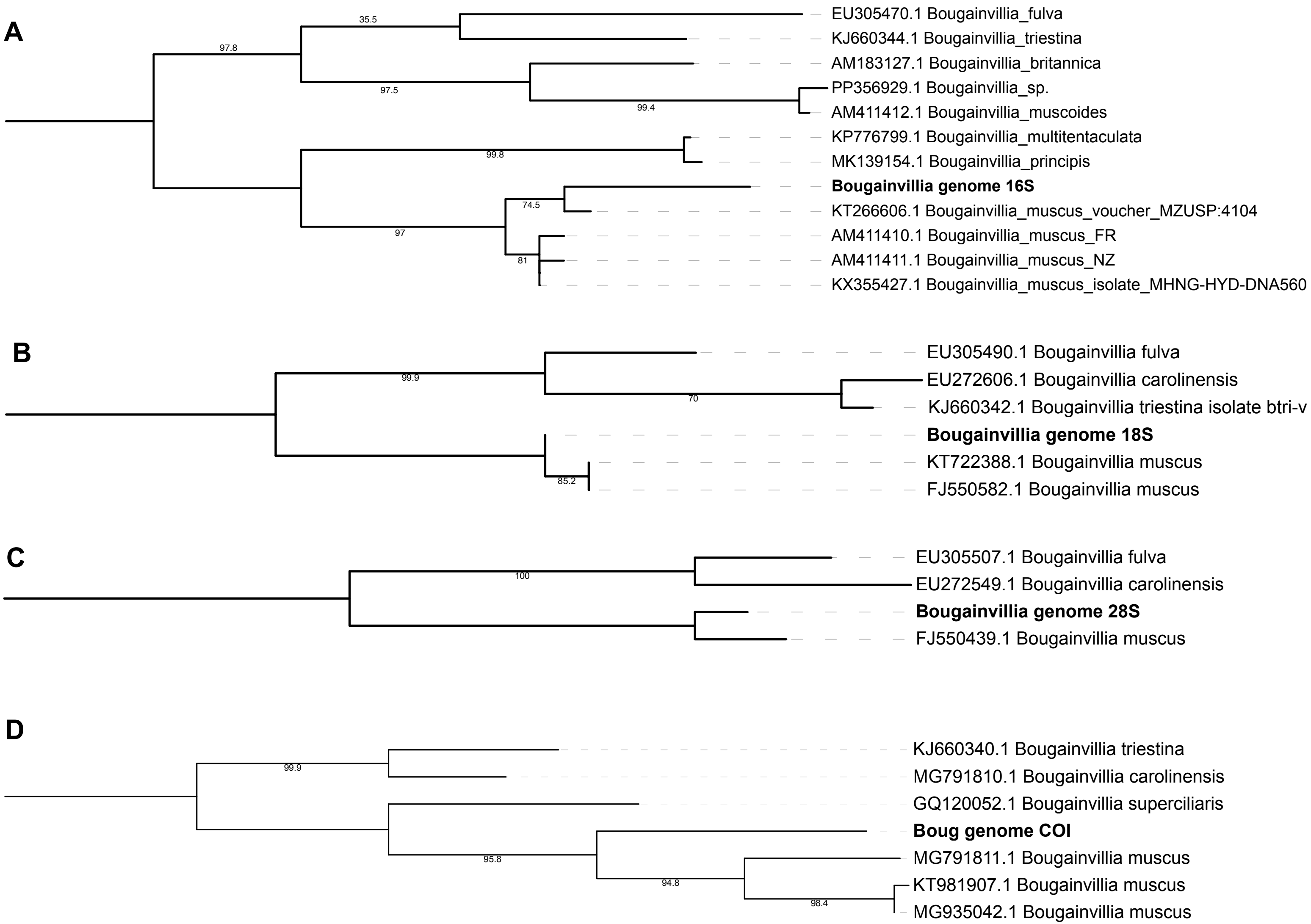

Figure S1. Bougainvillia 16S, 18S and 28S, and COI maker gene trees. A) 16S rRNA tree. B) 18S rRNA tree. C) 28S rRNA tree. D) Cytochrome oxidase subunit I tree. All trees are rooted at midpoint and show bootstrap support values.

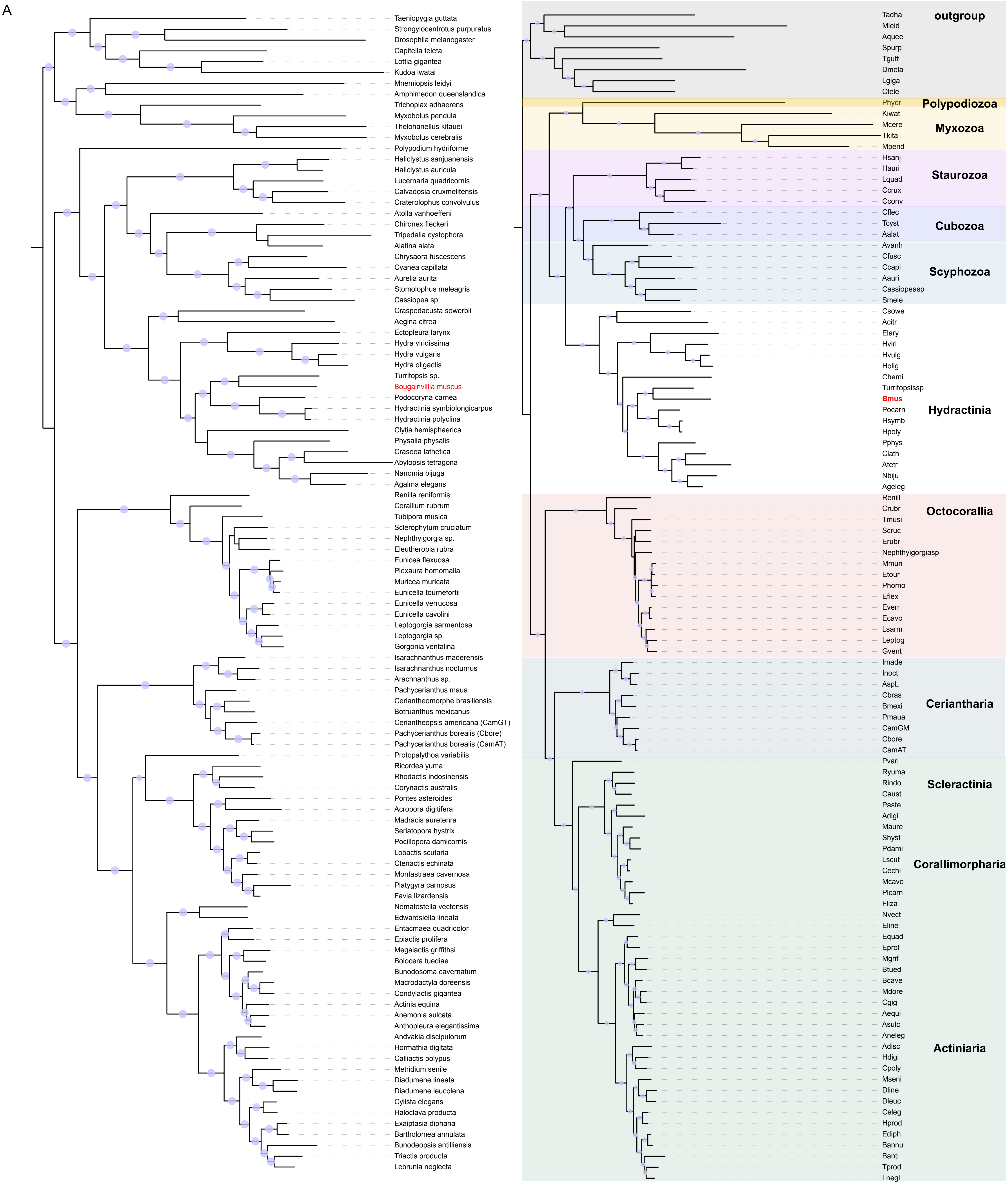

Figure S2. Cnidarian species-level tree. A) Phylogenetic tree generated using 748 untrimmed loci from DeBiasse. B) Phylogenetic tree generated using 748 loci and Gblocks, showing all branches.

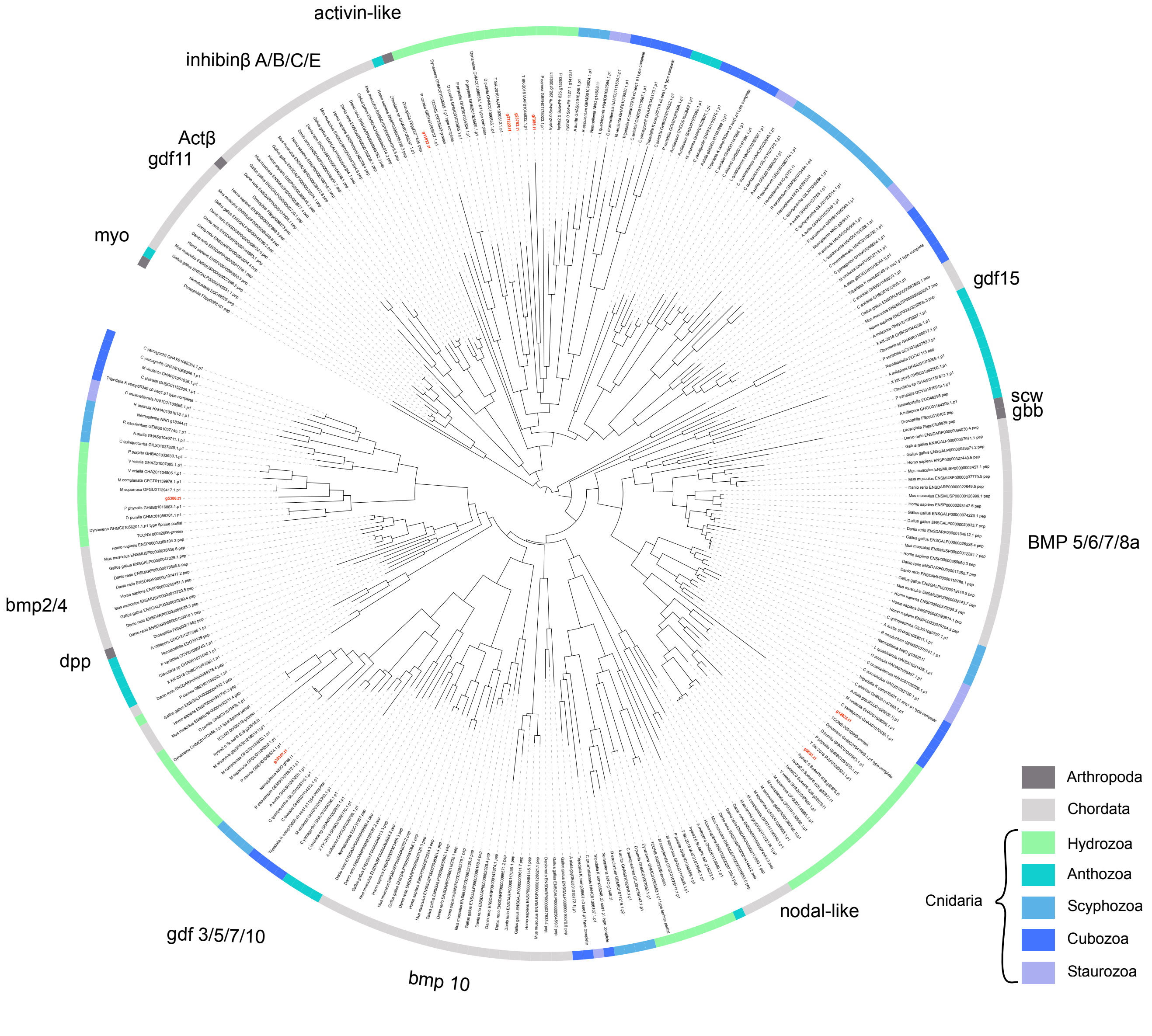

Figure S3. Growth Differentiation Factor (GDF) phylogenetic tree

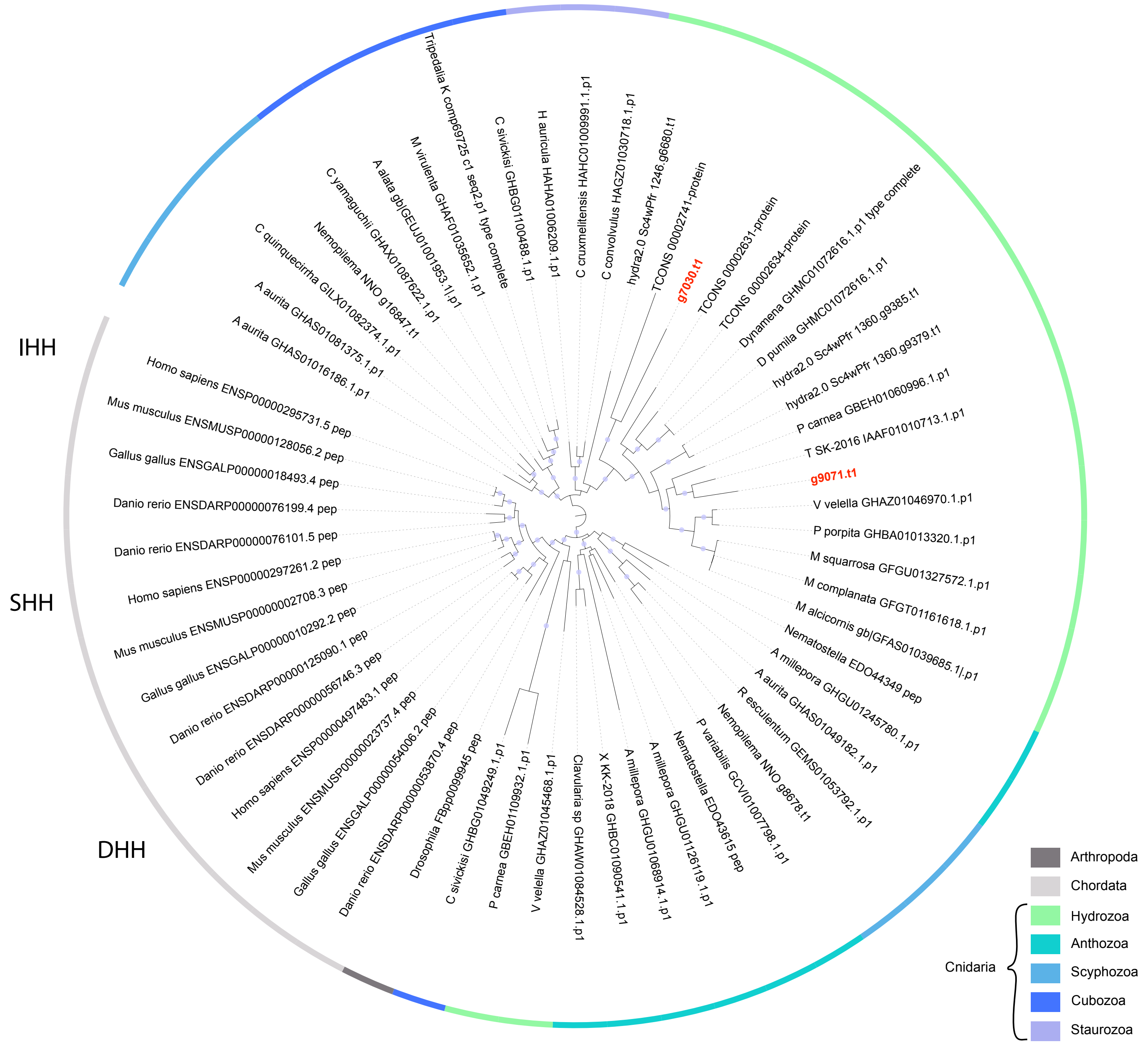

Figure S4. Hedgehog phylogenetic tree

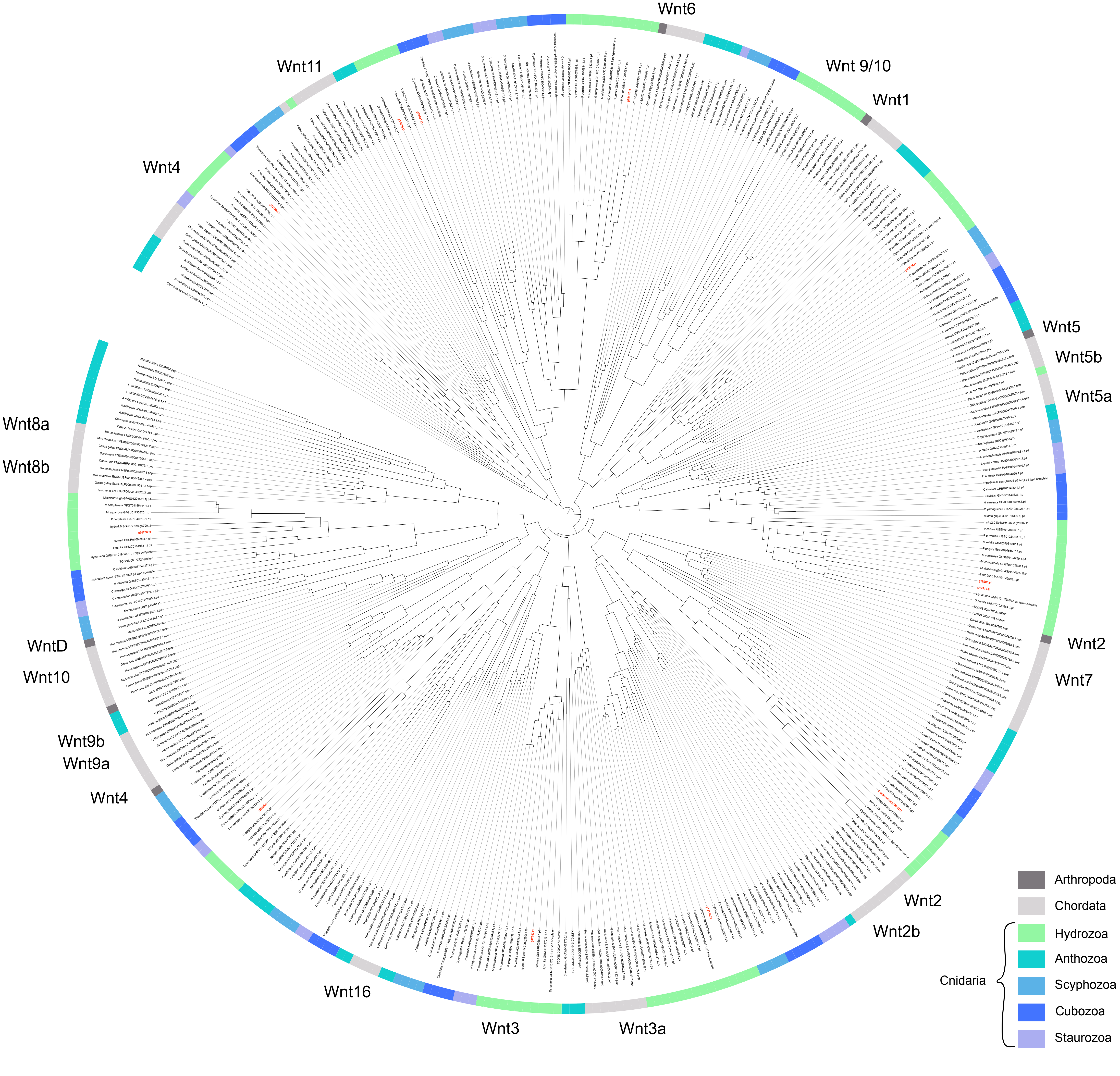

Figure S5. Wnt phylogenetic tree

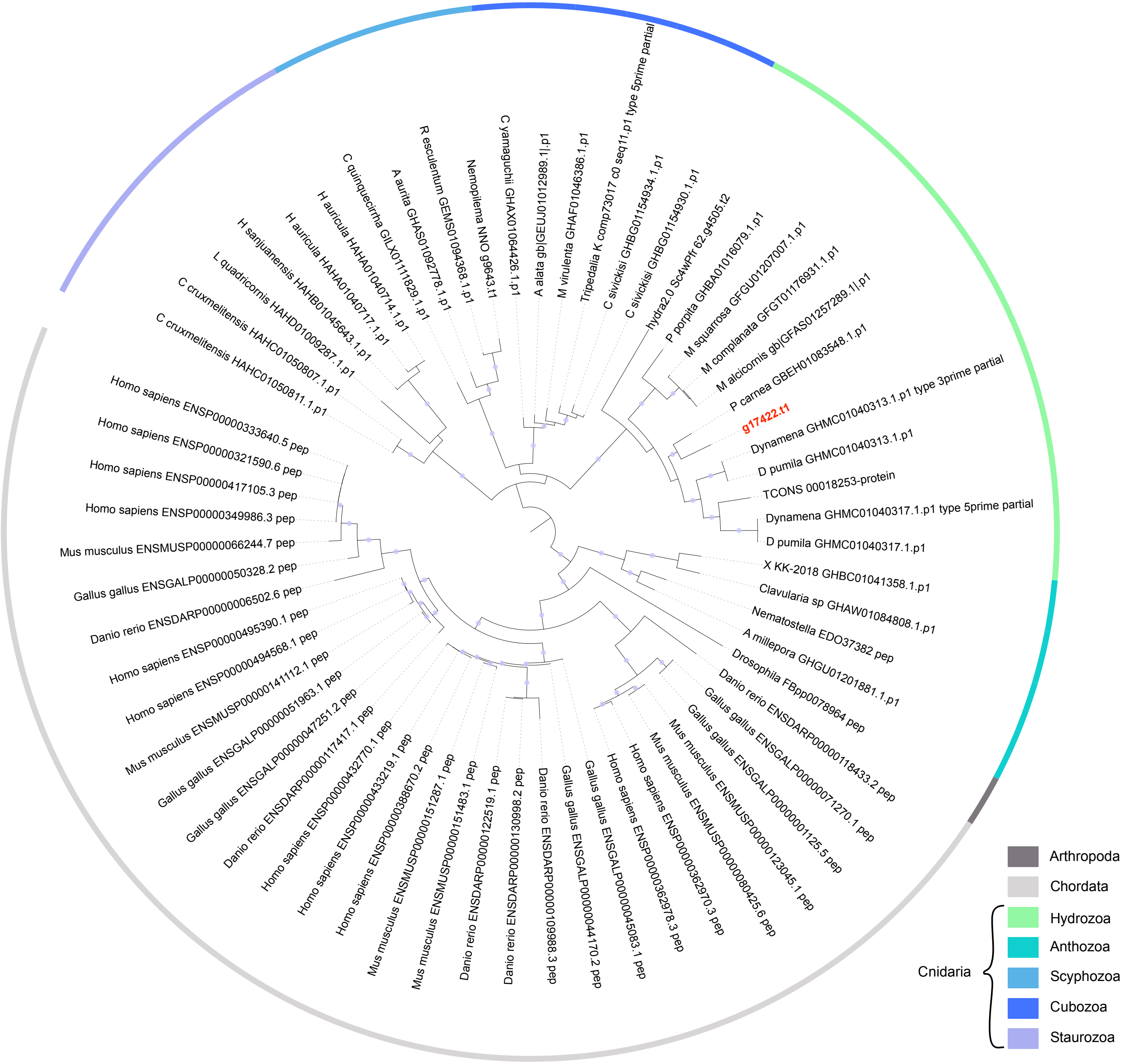

Figure S6. Eyes absent phylogenetic tree

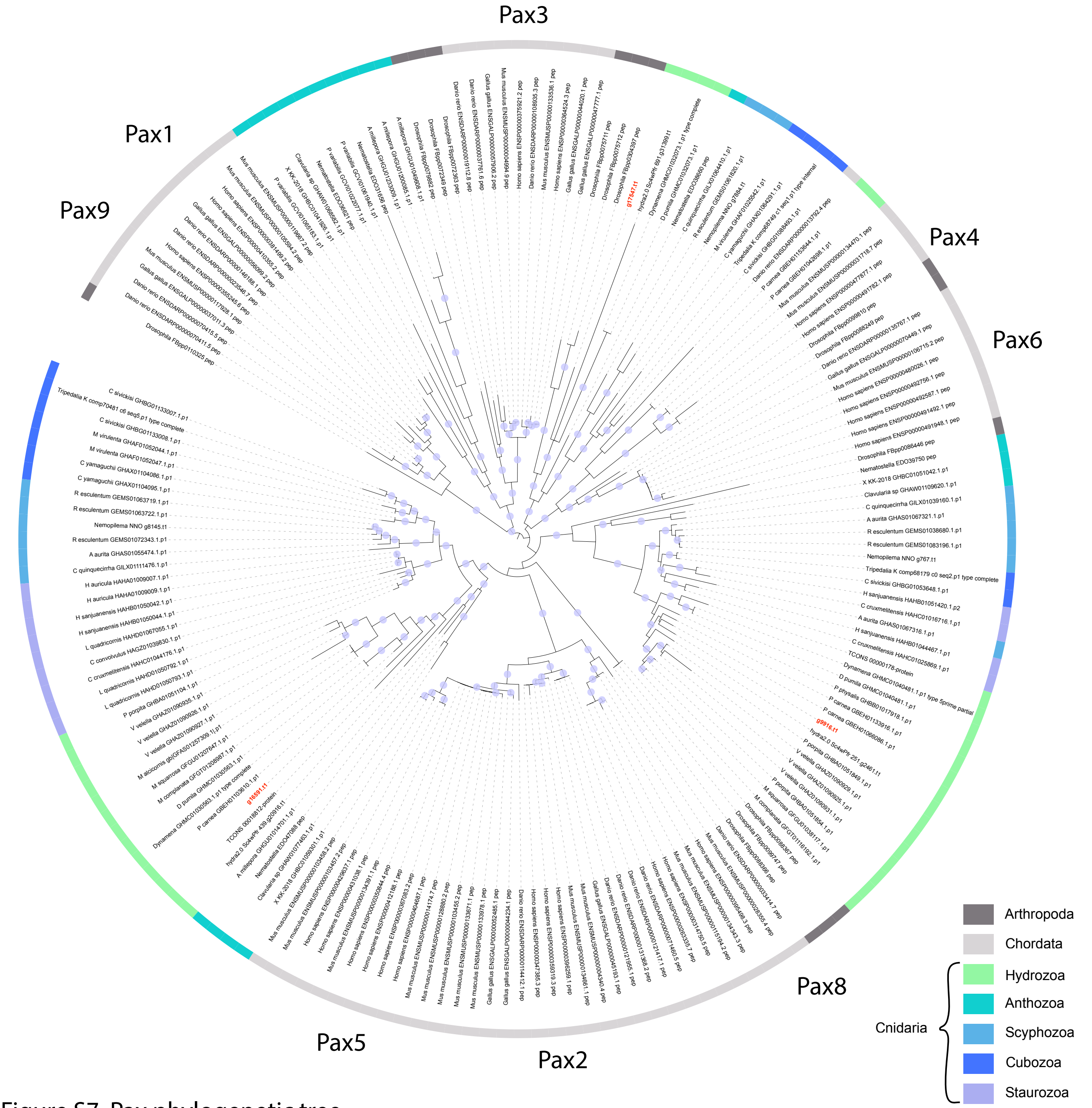

Figure S7. Pax phylogenetic tree

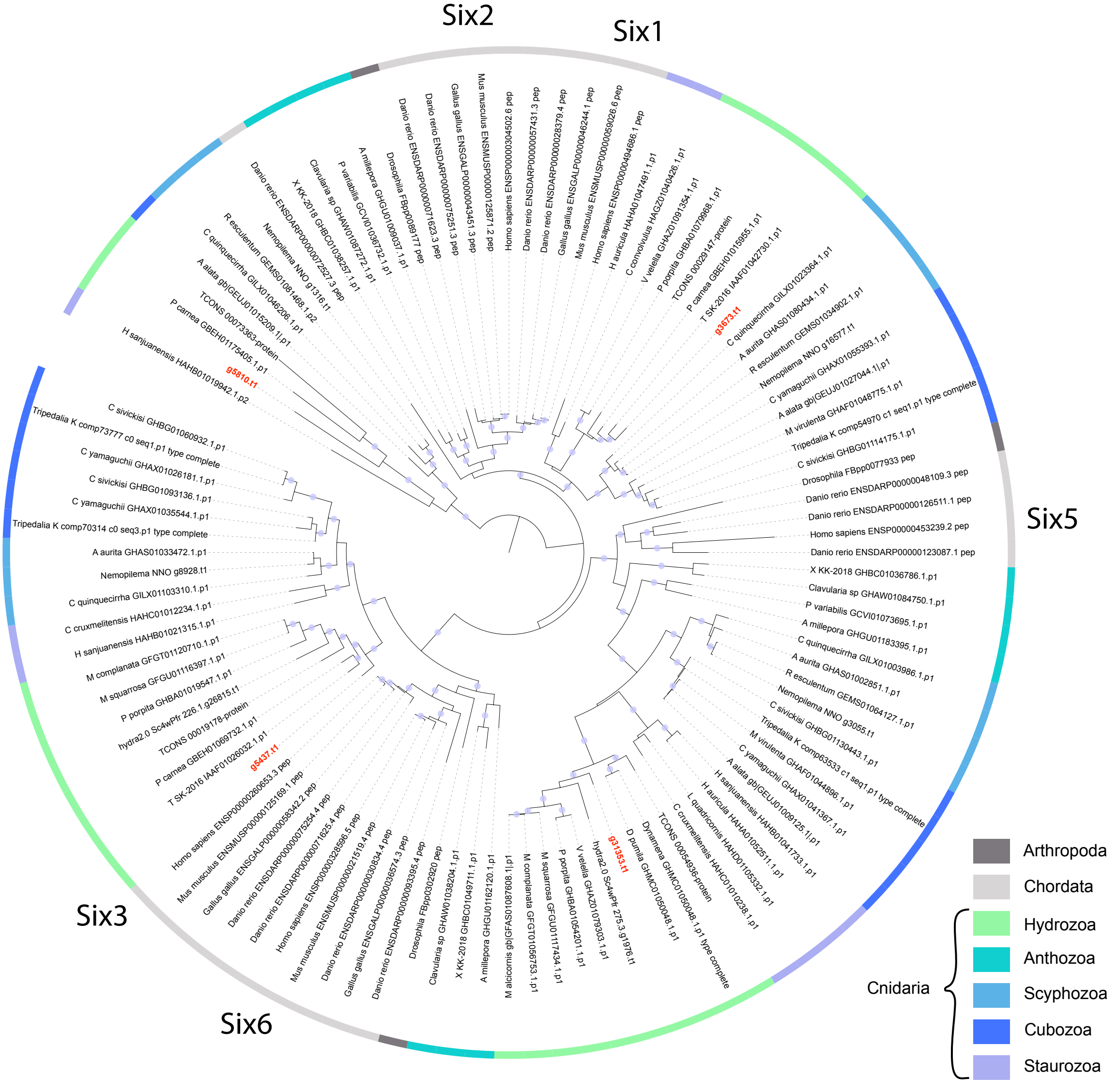

Figure S8. SIX phylogenetic tree

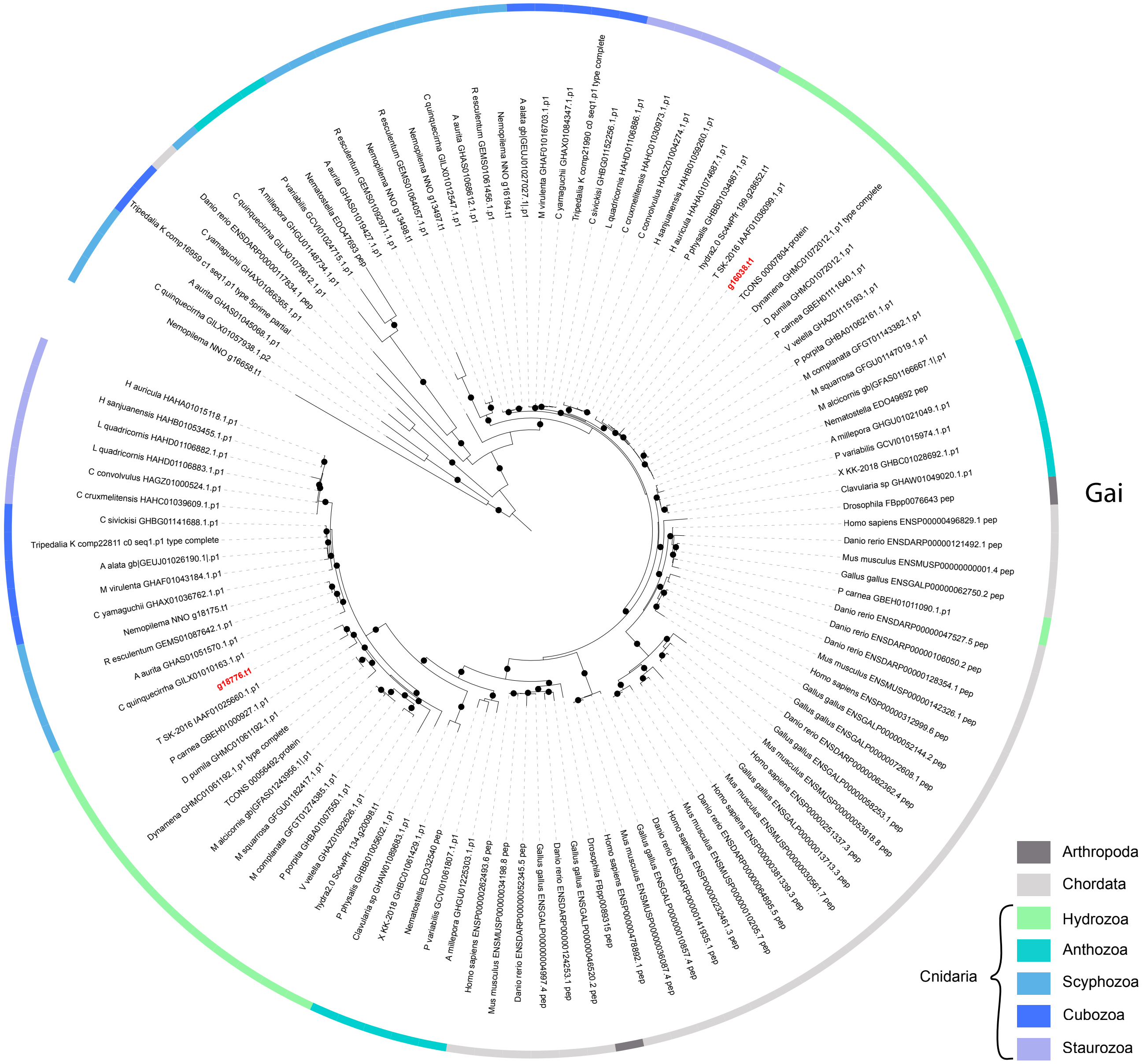

Figure S9. Galphai phylogenetic tree

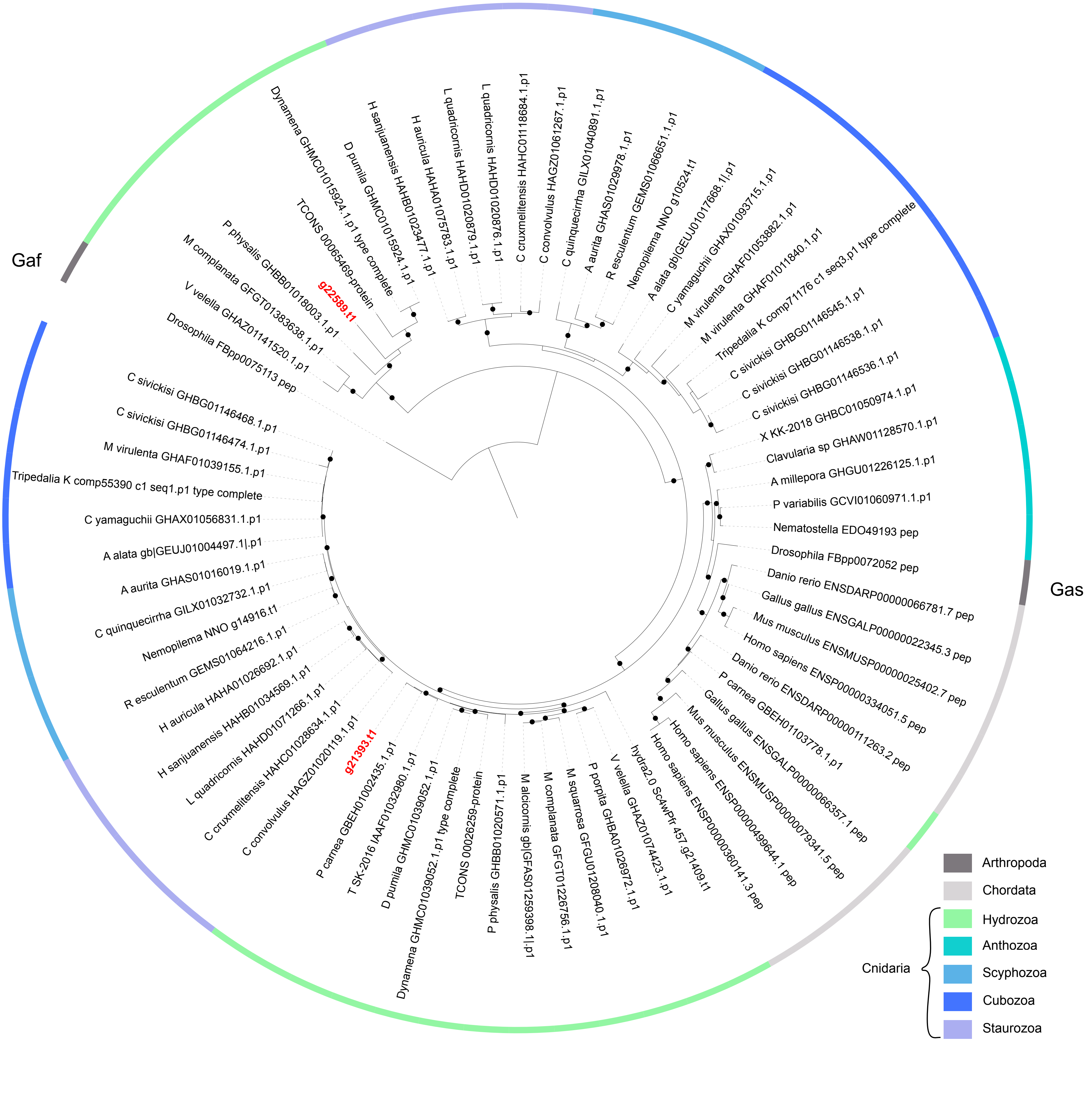

Figure S10. Gaplhas phylogenetic tree

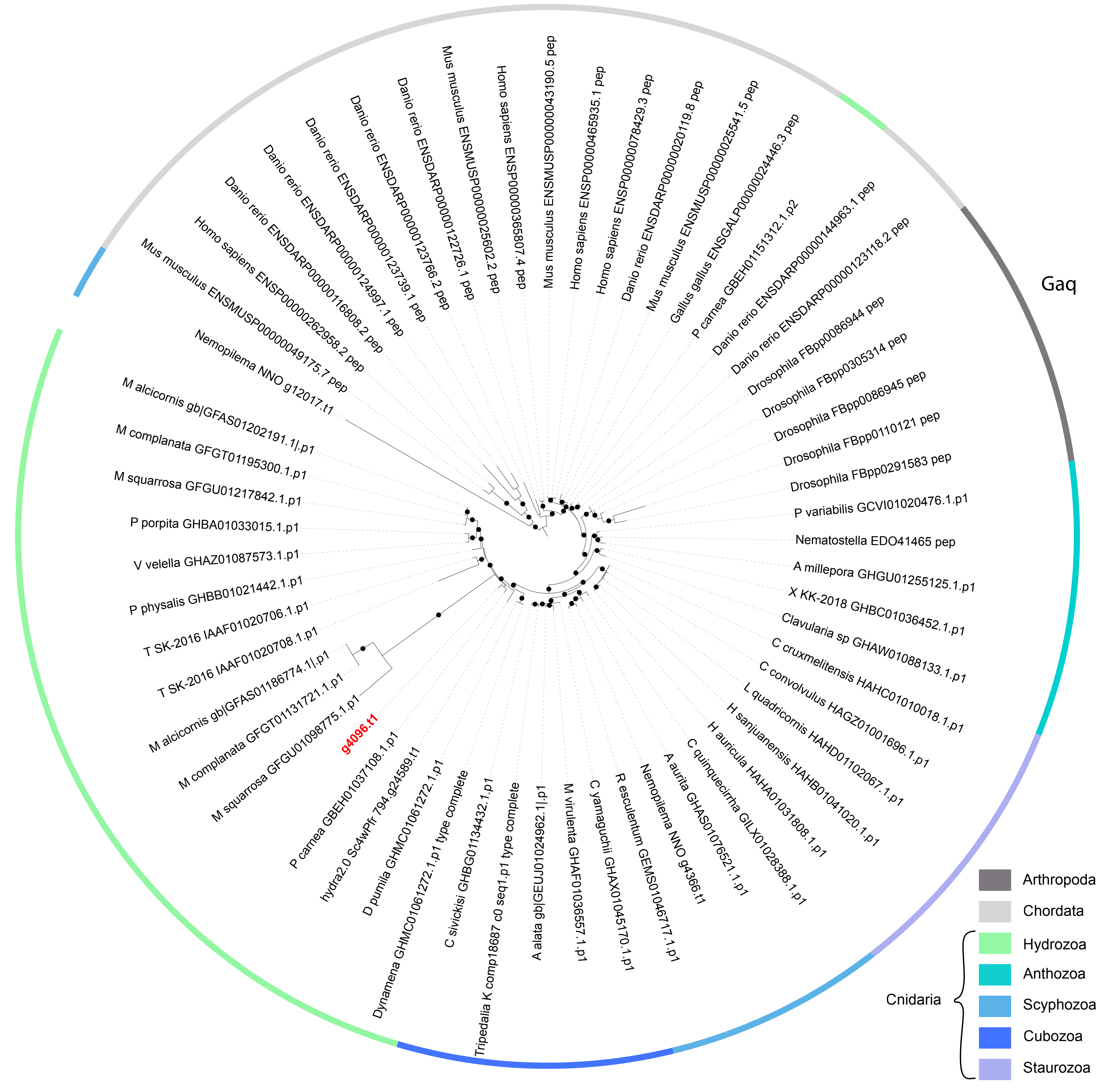

Figure S11. Galphaq phylogenetic tree

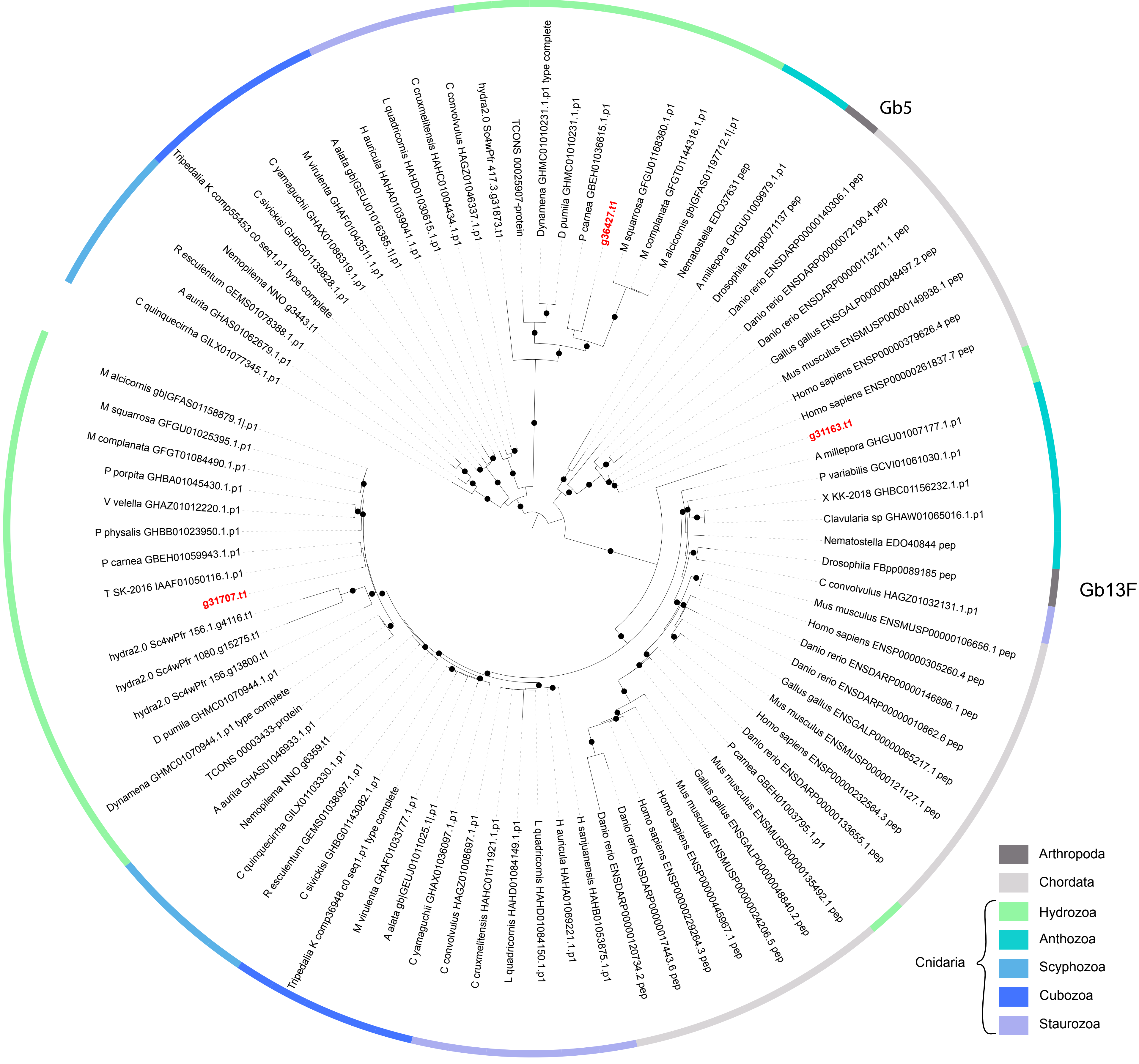

Figure S12. Gbeta phylogenetic tree

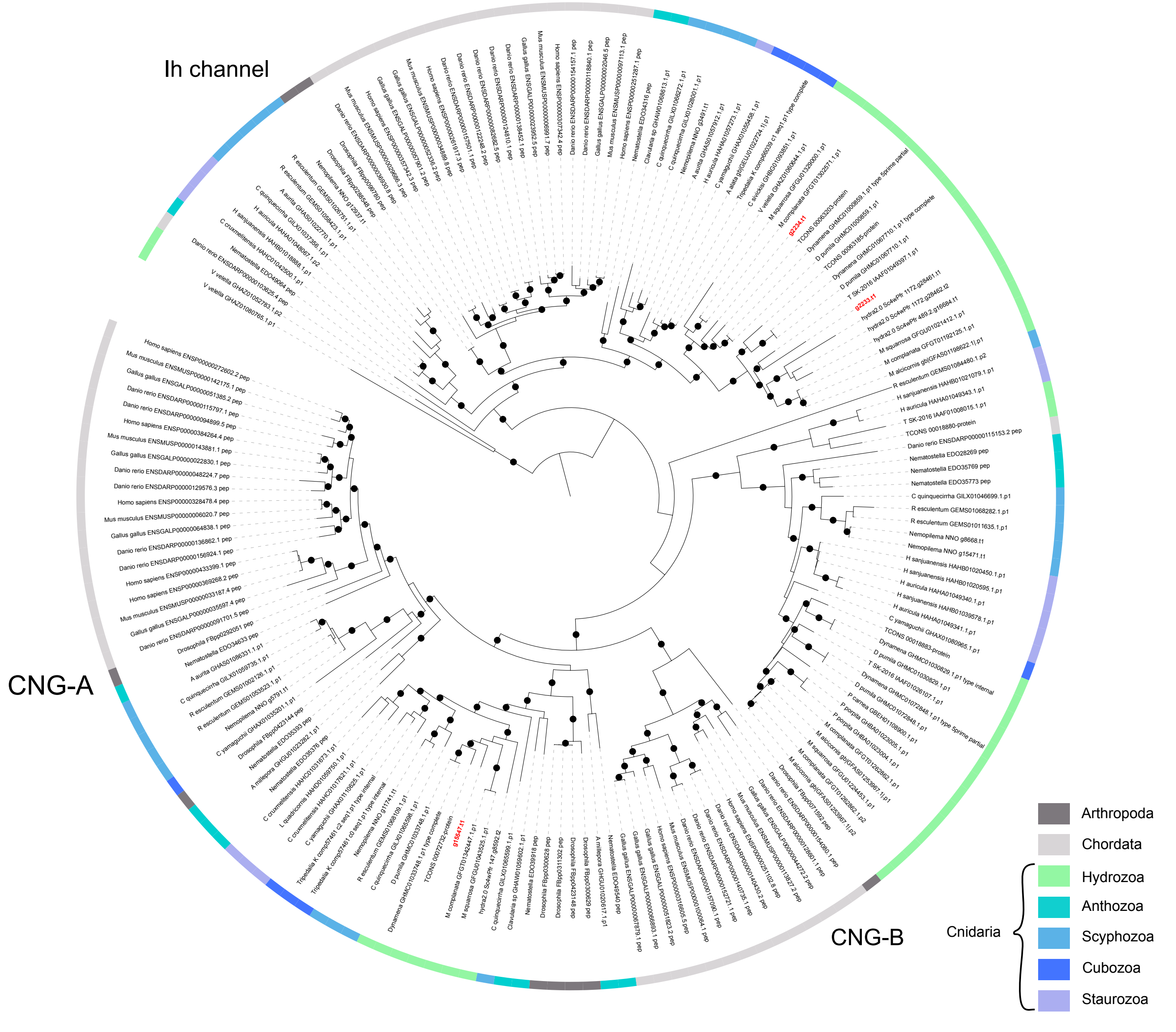

Figure S13. CNG phylogenetic tree

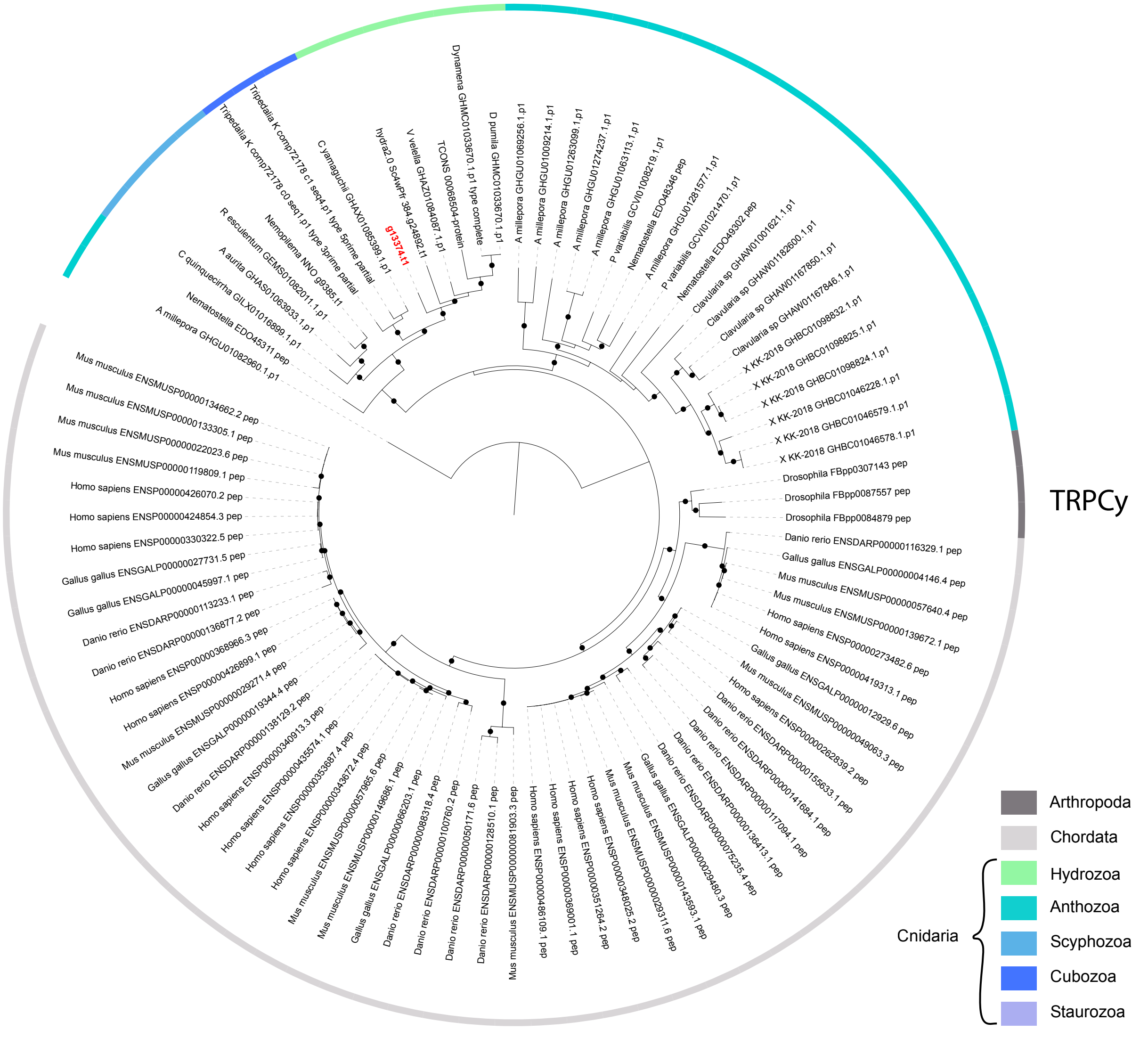

Figure S14. Transient Receptor Potential (TRP) phylogenetic tree

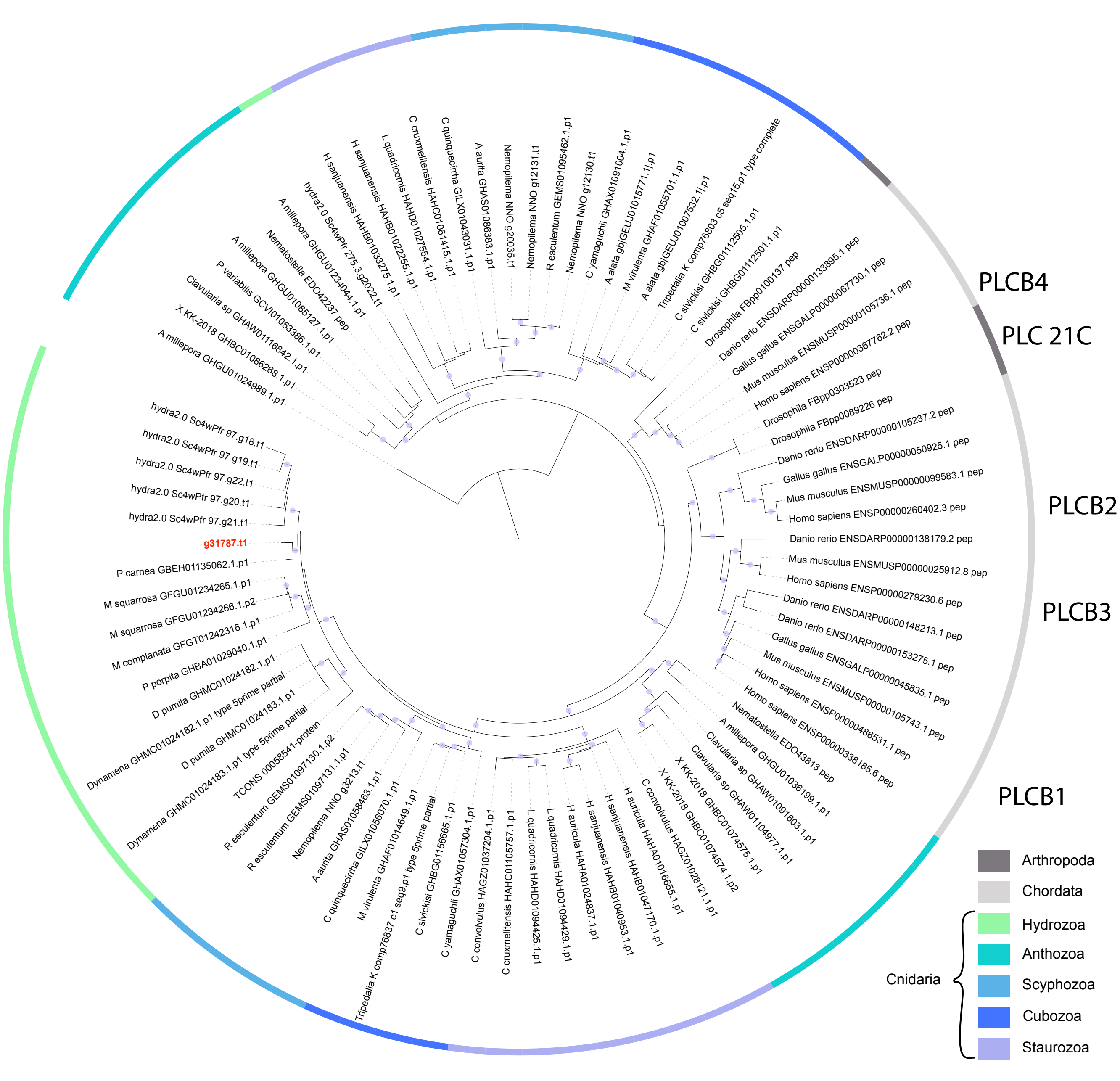

Figure S15. PLC phylogenetic tree

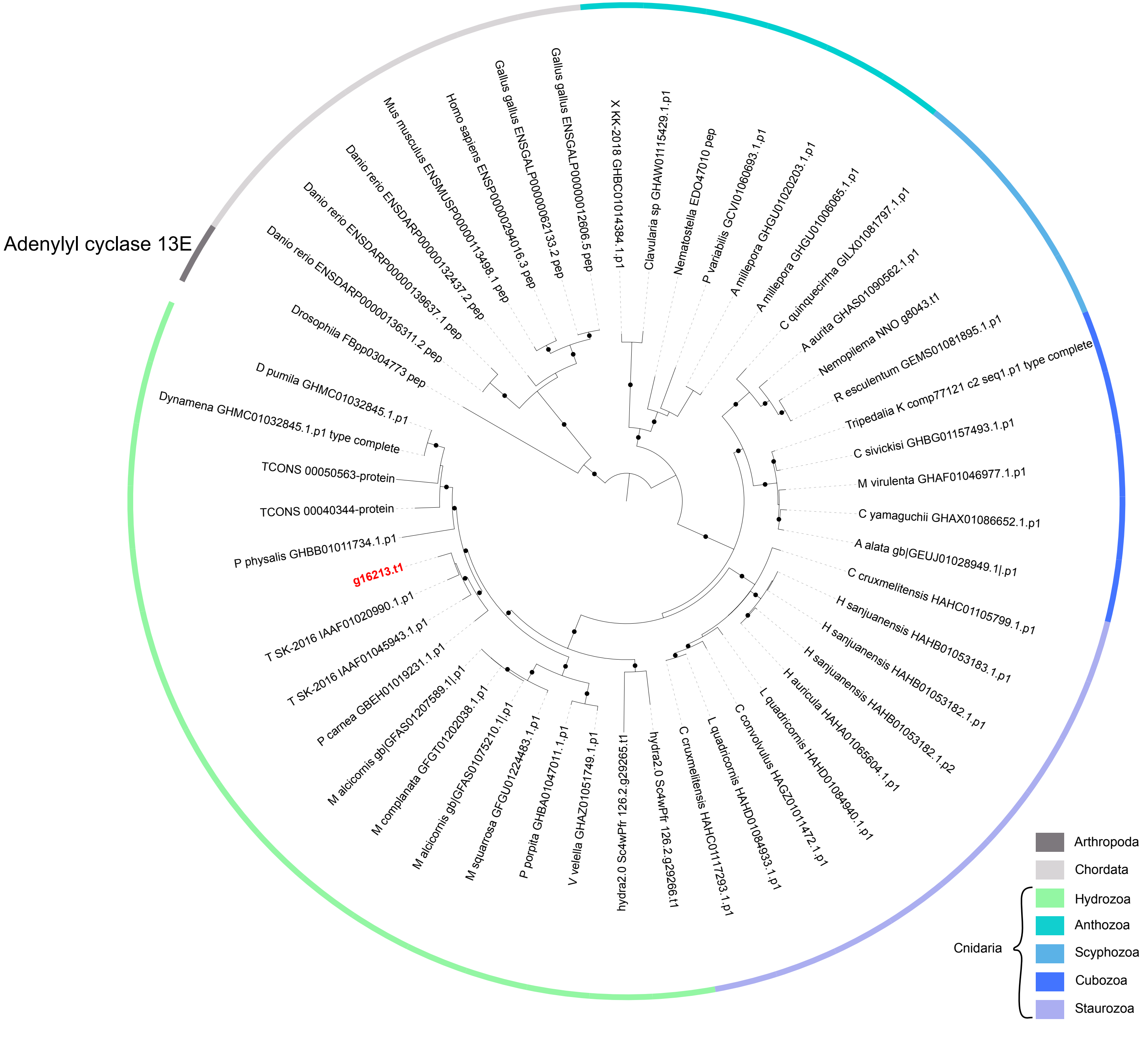

Figure S16. Adenylyl cyclase 13-like phylogenetic tree

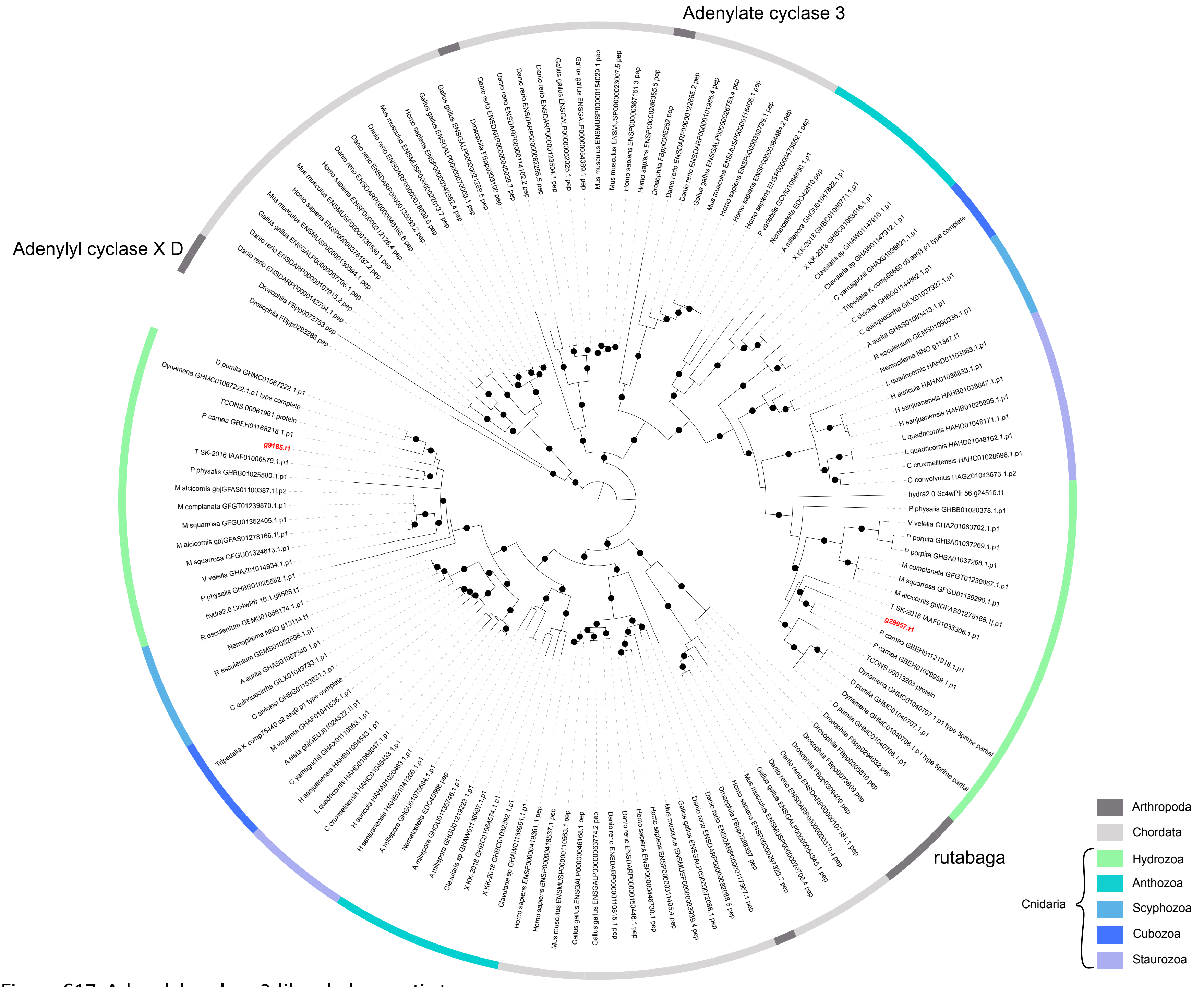

Figure S17. Adenylyl cyclase 3-like phylogenetic tree

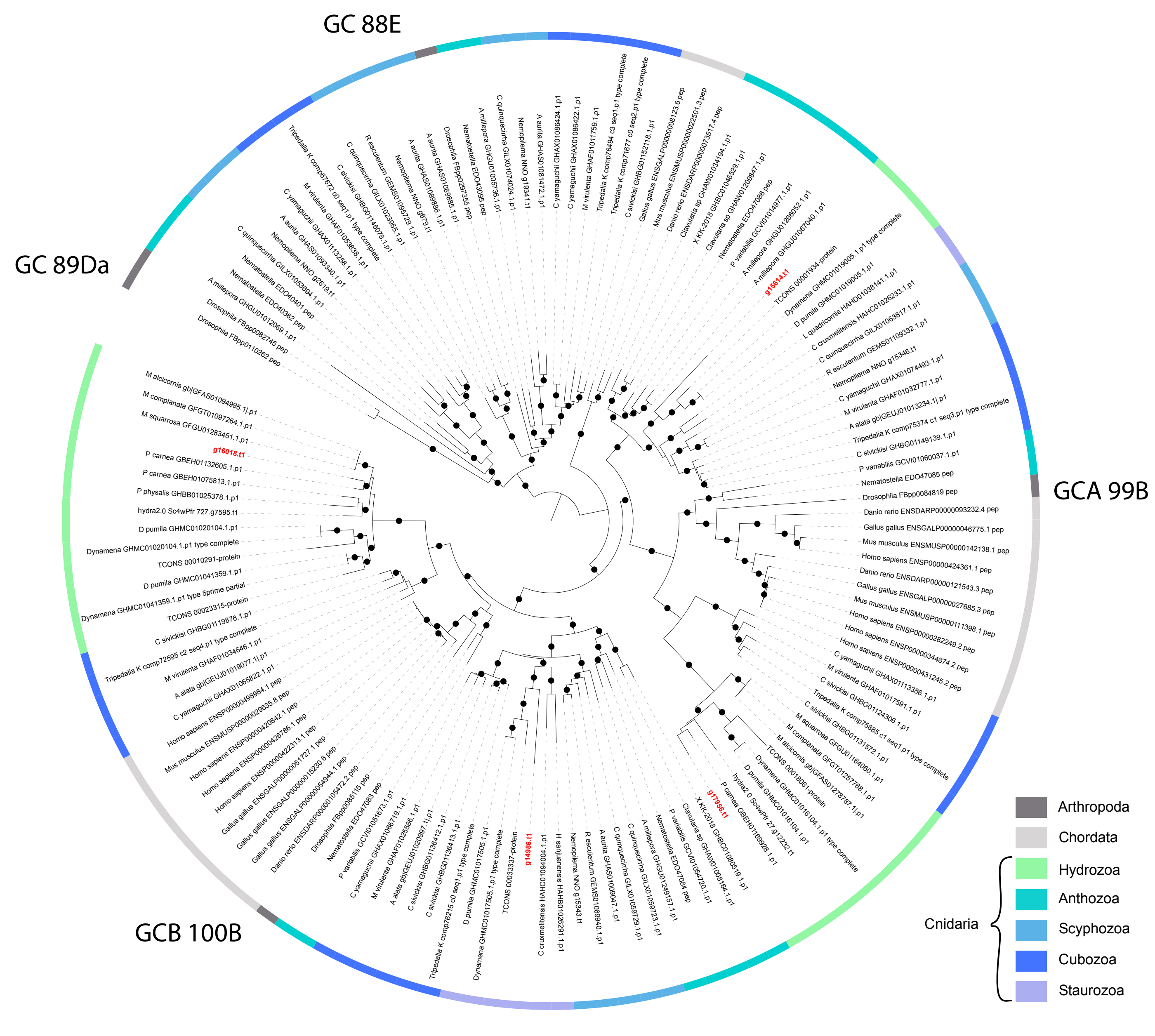

Figure S18. Guanylyl cyclase phylogenetic tree

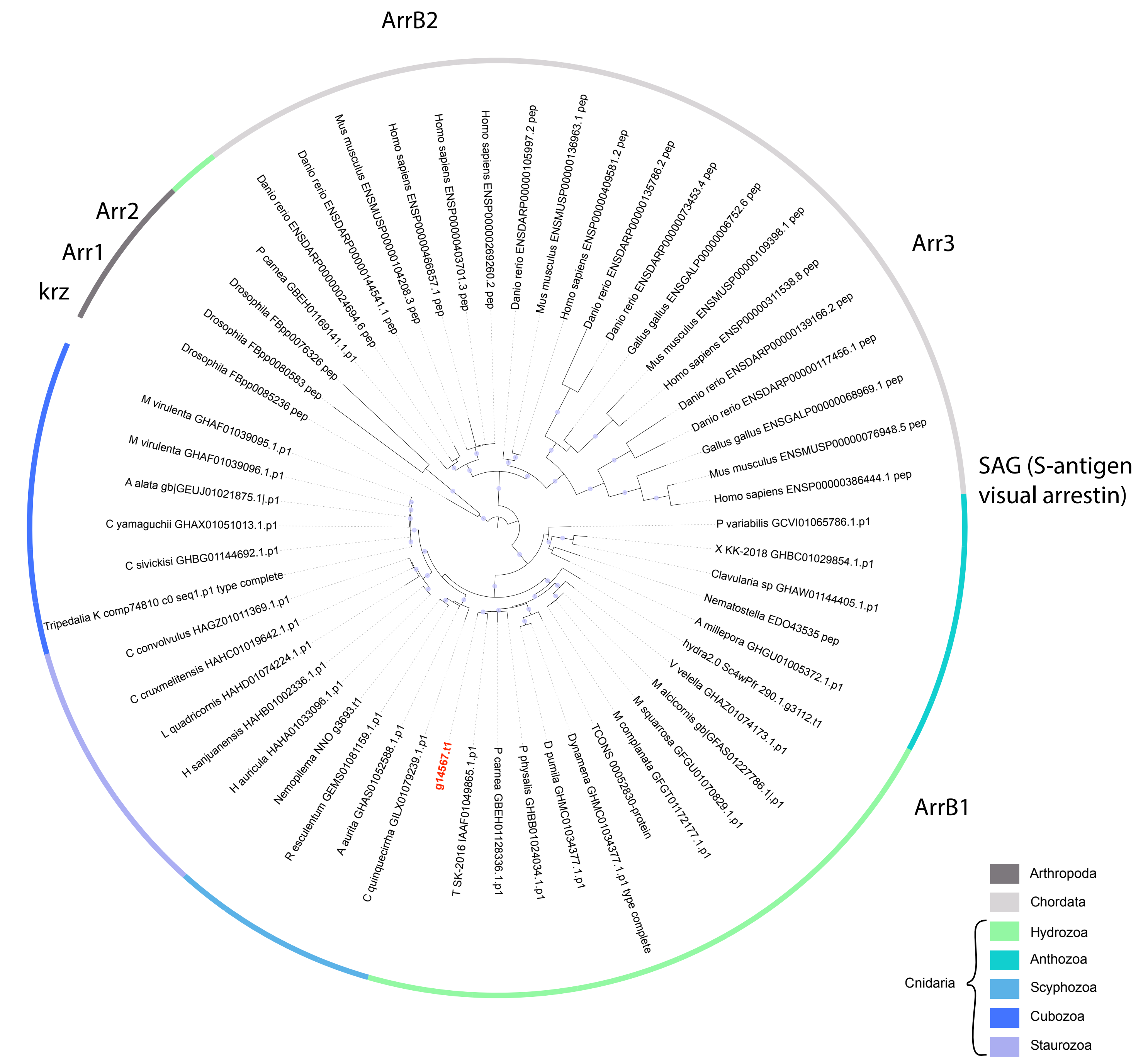

Figure S19. Arrestin phylogenetic tree

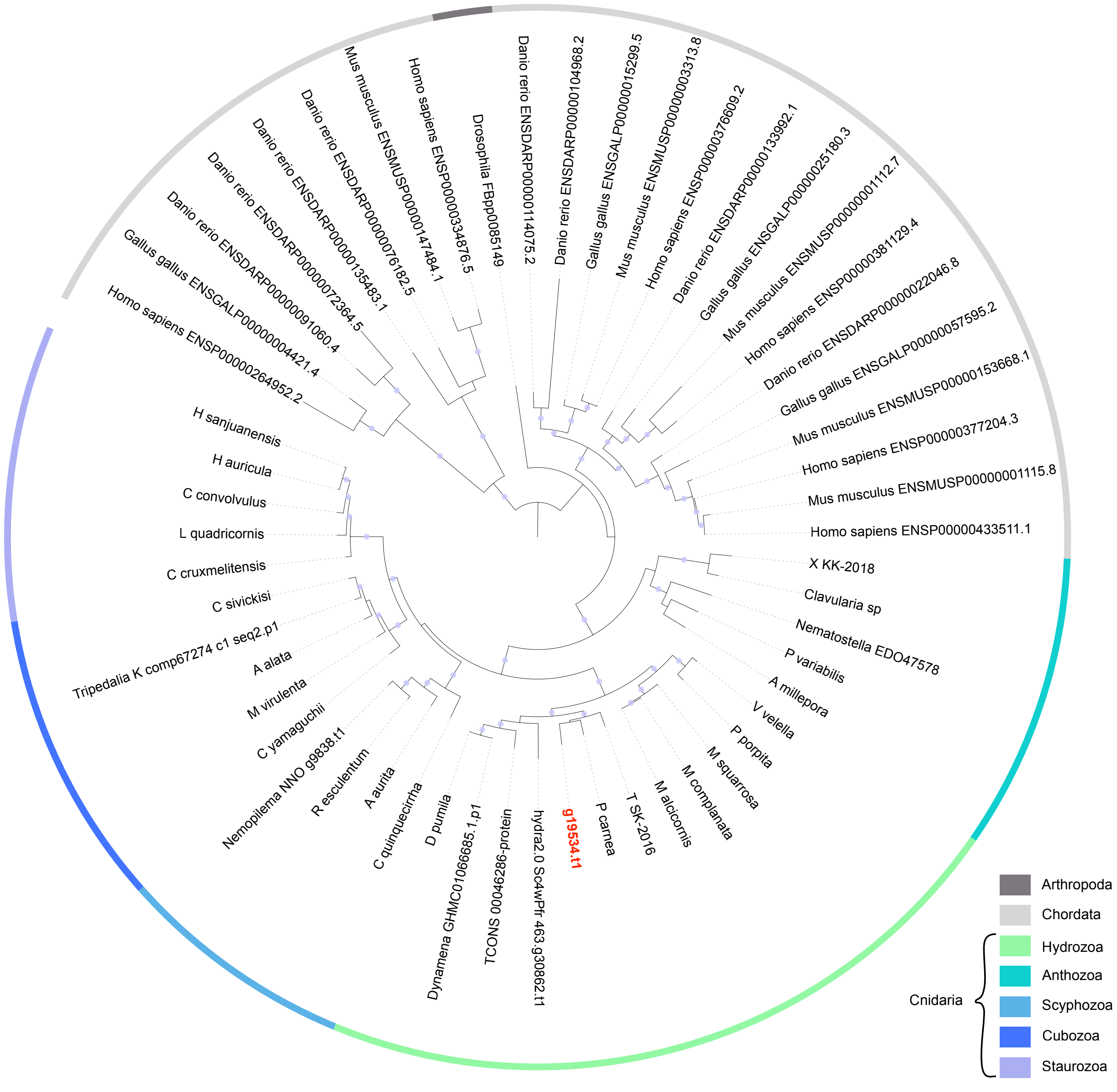

Figure S20. G protein-coupled receptor kinase (GRK) phylogenetic tree

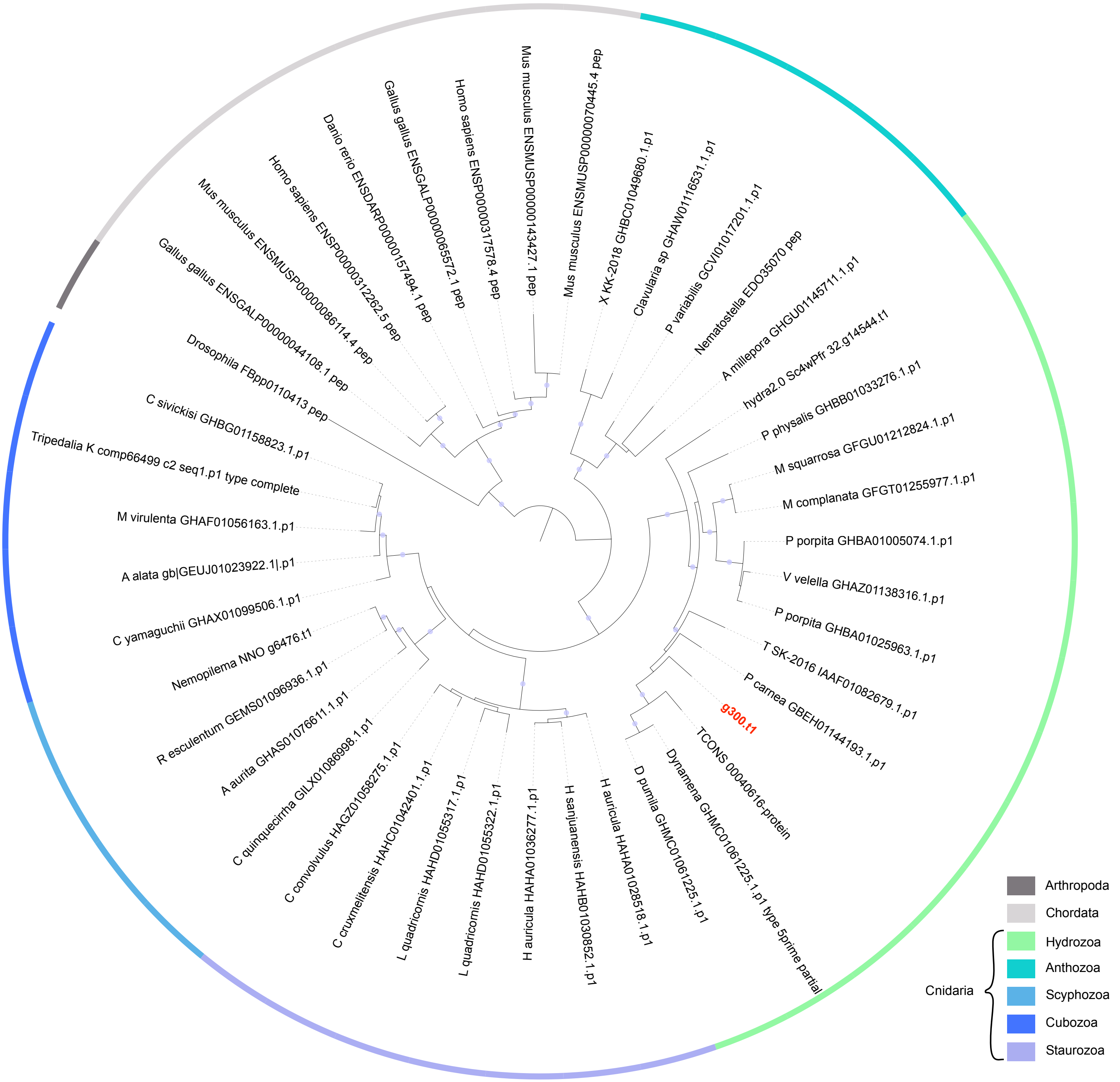

Figure S21. Rhodopsin kinase phylogenetic tree

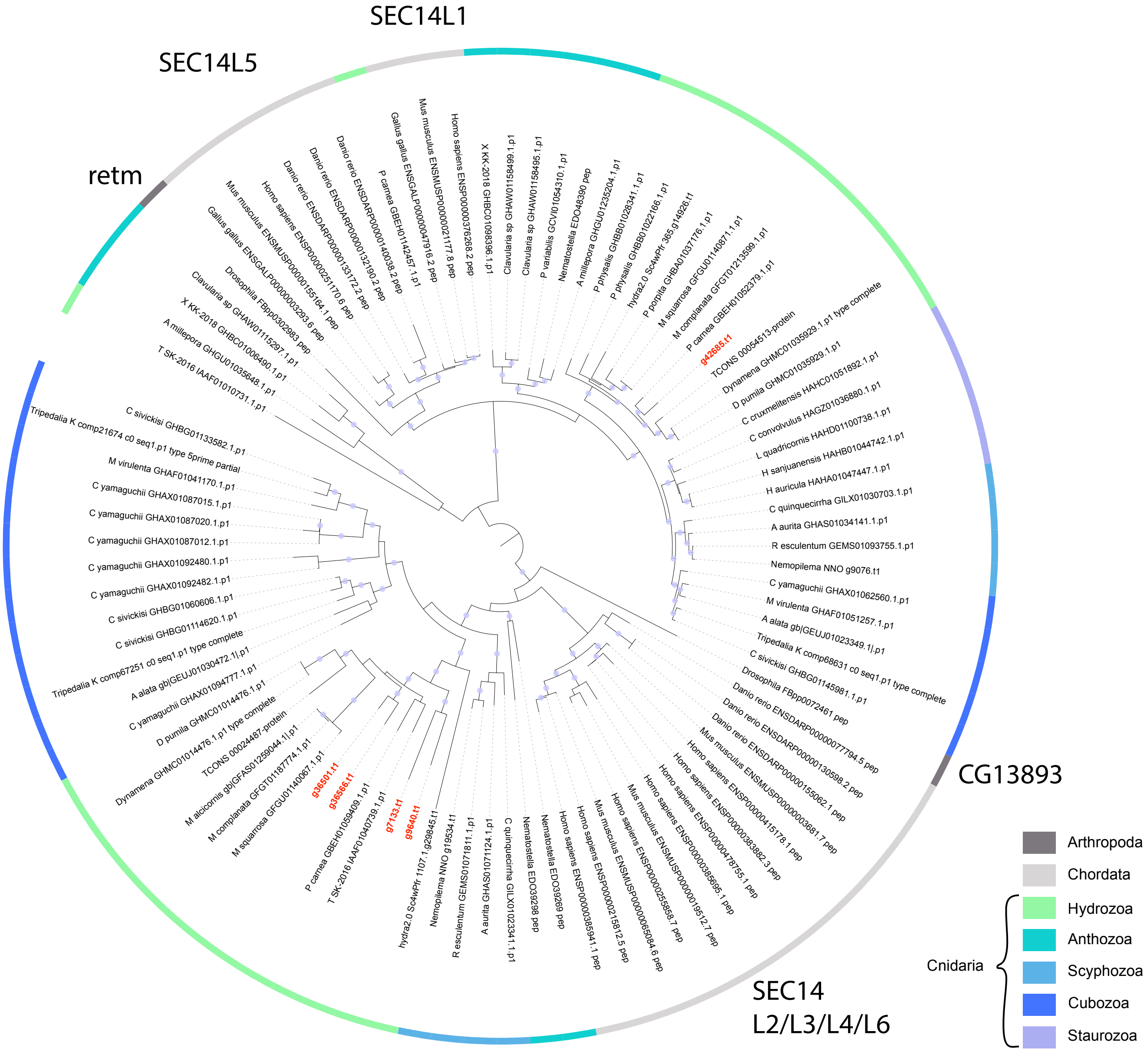

Figure S22. SEC14 phylogenetic tree

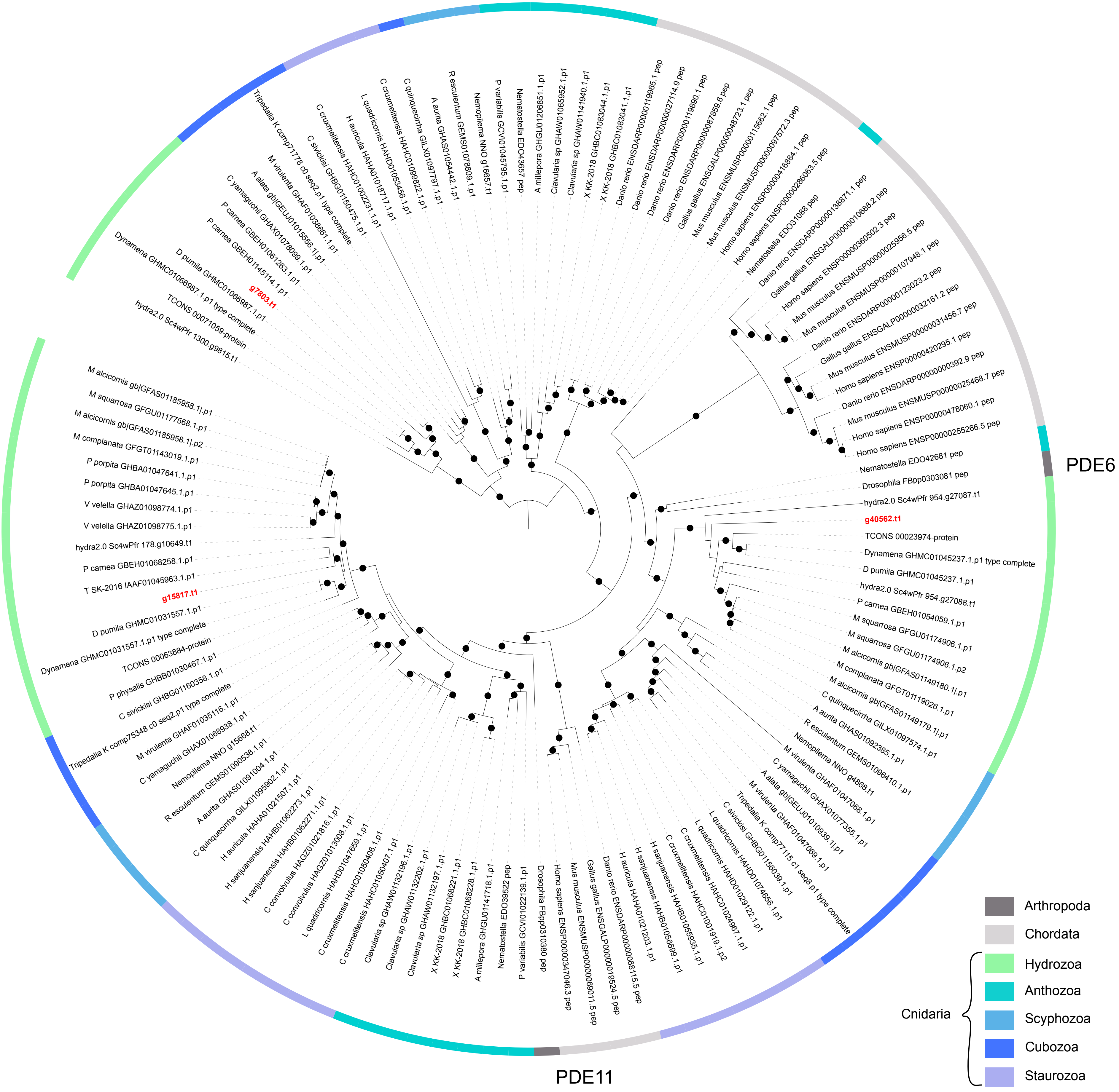

Figure S23. Phosphodiesterase phylogenetic tree
